# Cytotoxic plants from the Indian subcontinent: promising reservoir of anticancer agents

**DOI:** 10.5114/bta/221149

**Published:** 2026-08-20

**Authors:** Vipendra Kumar Singh, Vikas Kumar Singh, Urmilesh Singh, Aditya A. Singh, Ved Prakash, Rashmi P. Sharma, Ankit Kumar Singh

**Affiliations:** 1 Department of Biochemistry and Molecular Medicine, George Washington University, Washington, D.C., United States; 2 Department of Chemistry, Supaul College of Engineering, Supaul, Bihar, India; 3 Department of Botany, Institute of Science, Banaras Hindu University, Varanasi, Uttar Pradesh, India; 4 Department of Botany, University of Lucknow, Lucknow, Uttar Pradesh, India; 5 Zebrafish Research Laboratory, Department of Life Sciences, Parul Institute of Applied Sciences, Research and Development Cell, Parul University, Vadodara, Gujarat, India; 6 School of Life Sciences, Swami Ramanand Teerth Marathwada University, Nanded, Maharashtra, India; 7 University Department of Botany, Lalit Narayan Mithila University, Darbhanga, Bihar, India

**Keywords:** Indian subcontinent, cancer, anticancer drugs, medicinal plants, phytochemicals

## Abstract

In recent decades, cancer has remained the leading cause of mortality globally. The increasing incidence of cancer and the rise in the mortality rate pose substantial challenges for effective management strategies. Currently, several chemotherapeutic agents, including cisplatin, doxorubicin, and gemcitabine, are routinely used as frontline treatment options for cancer. Although these agents exhibit initial efficacy during the early stages of tumor development, their widespread application in clinical settings is significantly limited by their lack of selectivity, high cost, and toxicity to normal tissues. Phytochemicals have been recognized as promising alternatives to address these limitations. Their remarkable anticancer efficacy is attributed to various distinct mechanisms. Additionally, they are cost-effective and relatively nontoxic, which represent a significant advantage over conventional treatment approaches. To date, only a few phytochemicals, including resveratrol, gingerol, curcumin, and epigallocatechin-3-gallate, have progressed to clinical application. However, the current array of these phytochemicals is inadequate to meet the increasing demand for novel medicines against cancer. Thus, ethnopharmacology has emerged as a promising avenue for discovering and developing novel anticancer drugs. Medicinal plants from the Indian subcontinent are a valuable resource of diverse phytochemicals, which have traditionally been used for treating several health conditions and diseases. However, the evaluation of potential of these phytochemicals as anticancer medicines has largely been limited to preliminary screening. Therefore, this review specifically explores potent anticancer plants native to the Indian subcontinent that are yet to undergo rigorous clinical validation. It includes 60 plants from 44 families and details their therapeutic targets in various malignant cancers. It also discusses the major challenges and potential prospects for their effective application in cancer treatment.

## Introduction

In recent decades, cancer has consistently ranked among the leading causes of death worldwide (Sung et al. 2020). The GLOBOCAN database (2020) reported 10 million cancer-related deaths and 19.3 million new cases of cancer globally, with a predicted increase to 28.4 million new cases in 2040 (Deo et al. 2022). Various emerging and complex challenges are contributing to the cumulative risk of cancer and its related mortality worldwide. These include unhealthy lifestyle choices, exposure to pesticides through the food chain, premature aging, rapid population growth, and the emergence of new air pollutants, which significantly impact low socio-economic countries such as India (Cavalier et al. 2023). The alarming projection from the National Cancer Registry Programme 2020 highlights a critical public health crisis in India, with an estimated 2.08 million people expected to be diagnosed with cancer by 2040, marking an increase of 57.5% from the figures reported in 2020 (Ferlay et al. 2020). Additionally, in terms of cancer-related mortality, India ranks third, following China and the United States. The data also revealed that cancer remains the predominant cause of mortality in the 65+ age group globally, whereas it mostly affects the 40–64 age group in India (Ferlay et al. 2020; Sathishkumar et al. 2022). Many studies reported that the aggressive nature of cancer and its limited treatment choices, including radiation, chemotherapy, and surgery, have led to unsatisfactory outcomes (Anand et al. 2023). Currently, several chemotherapeutic agents, including anti-tubulin agents (taxanes), DNA interacting agents (such as doxorubicin and cisplatin), anti-metabolites such as methotrexate, hormones, and various other molecular targeting agents, are predominantly used in cancer treatment (Choudhari et al. 2020). However, resistance to chemotherapeutic agents, their severe side effects, tumor recurrence, and off-target efficiency pose significant challenges, which can negatively impact public health and socio-economic living standards (Wang et al. 2019). Thus, exploring new anticancer agents, particularly those derived from medicinal plants, is a promising avenue in cancer research because of their excellent therapeutic properties and minimal side effects (Tomar et al. 2024).

Phytochemicals, defined as compounds primarily derived from plants, are known for their therapeutic potential since ancient times (Dehelean et al. 2021). The unique characteristics of phytochemical compounds mostly rely on the diverse genetic constituents of plants and the distinct geographical environments in which they are grown (Moore et al. 2014). Moreover, several studies have reported that these phytochemicals can vary in chemical profile or quantity, even when extracted from the same plant species. Consequently, the development of new herbal medicines for cancer treatment has become a focus in all geographic areas (Lautié et al. 2020). To date, approximately 50,000 to 80,000 plant species have been explored for their potential medicinal applications (Chen et al. 2016). In this context, the National Cancer Institute (NCI) has undertaken substantial efforts to screen around 35,000 plant species to assess their viability for cancer treatment. The extensive screening yielded only 3,000 plant species that exhibited consistent anticancer activity (Desai et al. 2008). However, thus far, there are nearly 140 clinically approved antitumor drugs available for treating over 200 types of cancer. According to previous reports, almost 60% of widely prescribed plant-based anticancer medicines are derived from common sources, such as vincristine and vinblastine from *Catharanthus roseus*, paclitaxel from *Taxus brevifolia*, podophyllotoxin from *Podophyllum hexandrum*, and camptothecin from *Camptotheca acuminata* (Mazumder et al. 2020). Therefore, exploring untapped plant resources from various geographic locations for new anticancer compounds has been encouraged.

The Indian subcontinent is known for its biodiversity, encompassing four hotspots: the Indo-Burma region, the Western Ghats, the Himalayas, and the Sundalands. These regions host a rich array of medicinal plants, which have potential for contributing to the discovery of new anticancer agents (Regassa et al. 2022). Thus, the development of new anticancer therapeutics derived from medicinal plants, particularly those from the Indian subcontinent, has generated noteworthy interest within the field of contemporary cancer research. Despite the presence of several rich biodiversity hot spots and a blend of environments, phytochemical research in India remains in a nascent stage. Although various research groups are making valuable contributions to this field, there has been advancement only in few directions (Singh et al. 2023; Banerjee et al. 2016). Existing research is mostly conducted through *in vitro* experiments rather than through *in vivo* experiments. Therefore, extensive investigations into the pharmacokinetics and biodistribution of these molecules are necessary for validating their potential for cancer treatment.

## Plant-derived drugs: the Indian research scenario

Ancient scripture Ayurveda highlights the importance of plants as a prominent source of effective remedies for both acute and chronic diseases (Raghuvanshi et al. 2021). Additionally, the entire plant or its specific components, such as roots, stems, leaves, flowers, fruits, and seeds, are utilized in various forms, for example, dried powder, decoction, and herbal pastes, to treat a range of conditions, including infectious diseases, diabetes, neurological disorders, skin-related issues, and other health problems (Trivedi et al. 2024). This knowledge is extensively covered in textbooks such as *Ayurveda Materia Medica* and *Indian Materia Medica* (Gajarmal et al. 2020).

The Indian subcontinent has historically been a cultural and economic hub because of its favorable geographical characteristics. This has led to a rich heritage of plant-based traditional therapies, especially those rooted in Ayurveda and folk medicine (Jaiswal and Williams 2017). This proficiency arises from various factors, such as spiritual beliefs, easy accessibility, historical and cultural beliefs, and prevailing socioeconomic conditions. These practices thrived in remote areas due to a lack of modern medical facilities (Ravishankar and Shukla 2007). However, the practice of Ayurveda declined significantly during the medieval era due to political instability, external invasions, and the British colonial administration that focused on promoting allopathy through financial support (Jaiswal and Williams 2017). A renewed interest in Ayurveda emerged during India’s independence movement, prompting the government to implemente several key initiatives to protect these traditional medicine systems (Jaiswal and Williams 2017). In the mid-20^th^ century, research in plant-based therapeutics led to the discovery of vinblastine, vincristine, and podophyllotoxins, prompting the NCI to launch a plant collection program focused on temperate regions in 1960 (Kinghorn et al. 2016; Sak 2022). This initiative resulted in the discovery of various plant-derived compounds, including topotecan, irinotecan, navelbine, teniposide, etoposide, paclitaxel, and docetaxel, which are clinically approved as anticancer medications (Newman and Cragg 2020). Many herbs have been investigated for their ability to mitigate the side effects of cancer treatments and enhance immunity. These natural compounds often inhibit cancer cell growth by influencing cell proliferation, cell cycle regulation, apoptosis, angiogenesis, and metastasis (Dehelean et al. 2021). The commercial production of plant-based drugs has a slow pace of development with limited financial incentives, which hinders its progress. For instance, the development of effective clinical agents from camptothecins and taxanes spanned approximately 30 years, leading to a decline in pharmaceutical research interest (Li et al. 2017). However, the situation has changed significantly with the discovery of several new phytochemicals such as artemisinin, lupeol, curcumin, epigallocatechin-3-gallate, gingerol, resveratrol, and quercetin. Presently, these phytochemicals have reached the clinical application stage (Mazumder et al. 2020; Chandra et al. 2023). In 2015, the discovery of an artemisinin compound renewed hope regarding the potential of plant components to yield novel pharmaceutical drugs (Su and Miller 2015). A recent study showed significant progress in the past few years by examining new patents related to various polyphenols with anticancer properties (Gupta et al. 2023). Many plants used in ancient medicine have increasingly become an integral part of the modern healthcare system. Consequently, this traditional knowledge of medicinal plants has evolved into a highly profitable industry, with estimates suggesting that the global demand for therapeutic plants might reach $5 trillion by 2050 (Koul and Koul 2019). The Indian subcontinent is known for its safe and effective ethnomedical research practices (Shi et al. 2021). Ancient remedies were developed based on practical experience rather than in-depth scientific understanding of their effective ingredients (Pandey et al. 2013). Recently, Indian research groups have focused on exploring the anticancer potential of numerous plants, primarily through plant extracts rather than investigating isolated compounds (Reddy et al. 2023). Thus, this review aims to present information regarding cytotoxic plants from the Indian subcontinent, highlighting preclinical reports on their *in vitro* activity and tests on animal models for various cancers (Figure 1).

**Figure 1:**
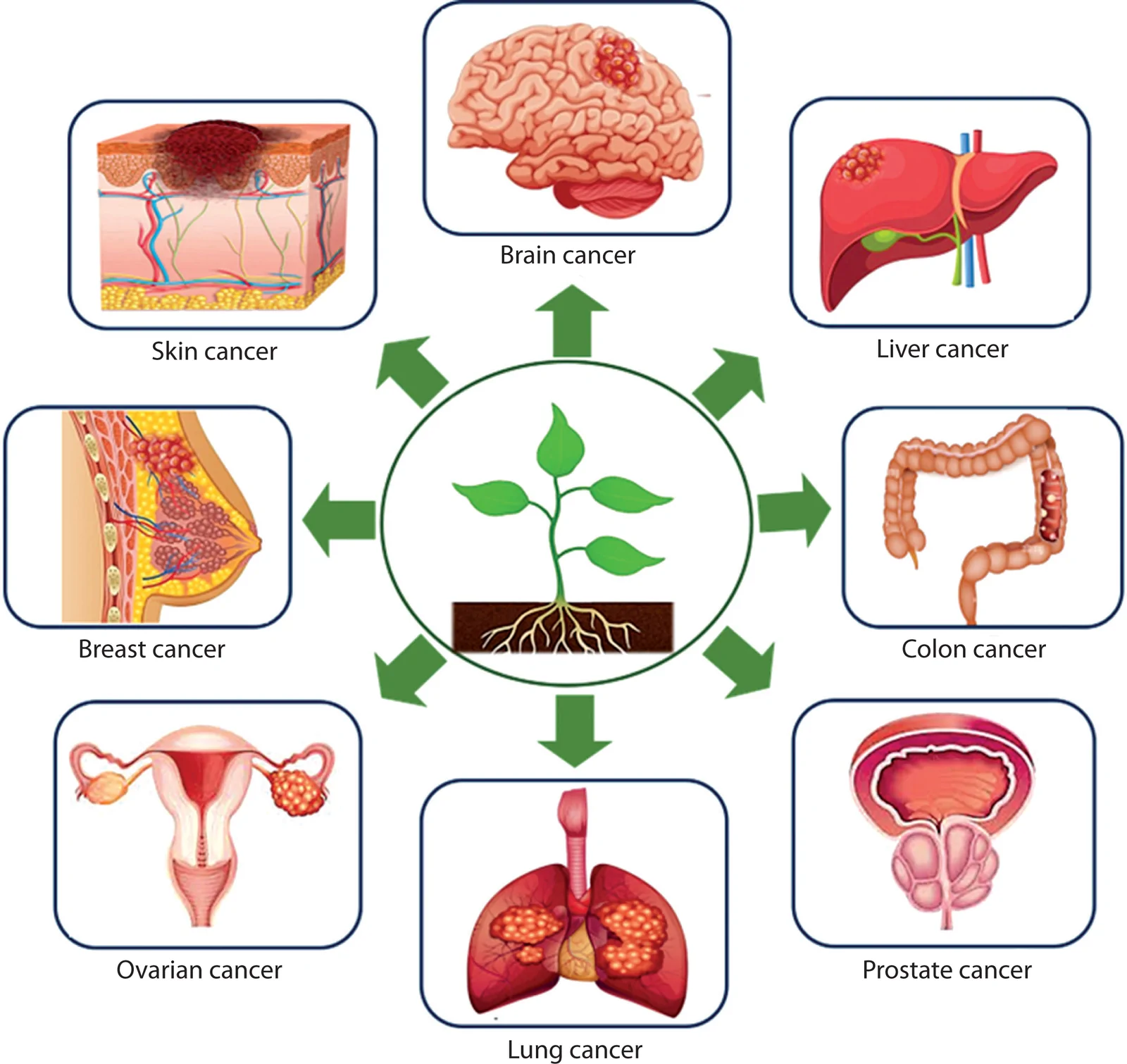
Cytotoxic plants showed anticancer potential in many types of cancers

## Potential cytotoxic plants from the Indian subcontinent

Ayurveda, the oldest medical system in India, continues to exert substantial influence on current medical practices and has contributed significantly to advancements in global healthcare (Fatima et al. 2021). Presently, the most commonly prescribed medications such as aspirin, morphine, digitoxin, artemisinin, and quinine are mainly derived from ethnomedicine practices. While numerous Indian plants are noted for their remarkable therapeutic benefits, their cytotoxic properties have often overlooked (Prasathkumar et al. 2021). Nevertheless, vital knowledge about Indian cytotoxic plants is preserved in sacred texts, awaiting further discovery. A narrow focus on known anticancer plants limits our understanding of less-studied species. Thus, this review examines lesser-known plants for their potential anticancer compounds. Some of the significant plant-based bioactive compounds with anticancer potential are summarized in Supplementary Table 1, with their possible effects illustrated in Figure 2 and succinctly reviewed below.

**Figure 2:**
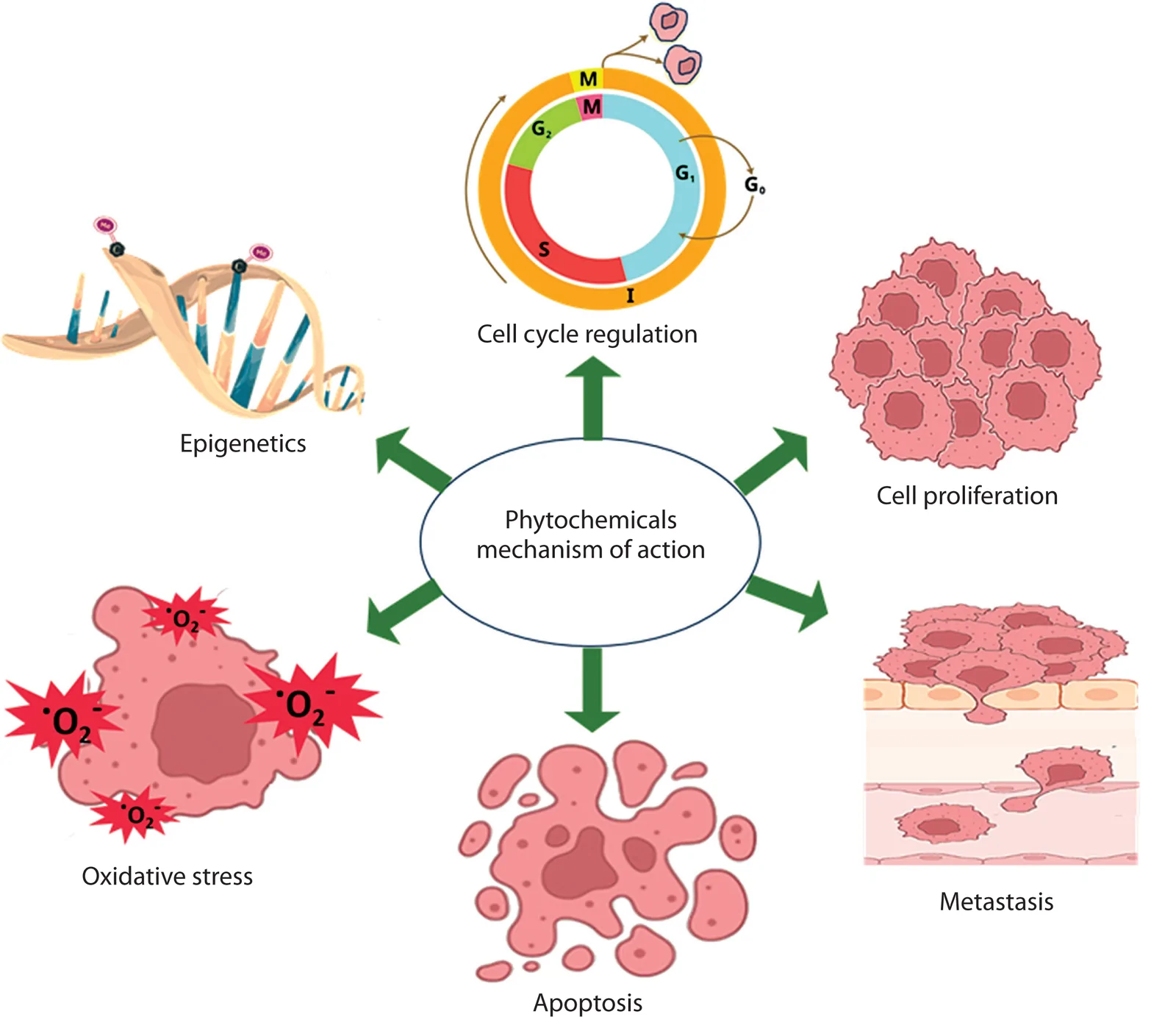
Phytochemicals and possible mode of action

### *Abrus precatorius* L.

*Abrus precatorius* L. is a tropical medicinal plant in the *Fabaceae* family. Generally, its seeds, roots, and leaves are used to treat various disorders such as parasitic worms, diarrhea, depression, cataract, allergies, cancer, and infertility. Recent studies have shown that its bioactive components, tannic acid and rutin, exhibit promising anticancer activity against cervical carcinoma Hep-2C and HeLa cells and act as cyclooxygenase-2 inhibitors (Kaur et al. 2024). Isoflavanquinones derived from the roots prevent inflammation by promoting phagocytosis and inhibiting the expression of the cytokine tumor necrosis factor-alpha (TNF-α). Additionally, abruquinones M and N exhibited cytotoxic effects against the human colorectal adenocarcinoma (Caco-2) and human tongue squamous cell carcinoma (CAL-27) cancer cell lines, with abruquinone M also suppressing the proliferation of the human non-small cell lung cancer cell line (NCI-H460) (Okoro et al. 2021).

### *Adenanthera pavonina* L.

*Adenanthera pavonine* possesses several therapeutic properties, including astringent, antioxidant, anti-inflammatory, and anticancer effects. It is used to treat gout, rheumatism, diarrhea, and hypertension (Krishnan et al. 2022). A previous study revealed that its seeds and leaf extracts are effective against various pathogens and cancer cells (Chauhan et al. 2015). Another study showed that the chloroform extract of the leaves significantly inhibited the growth of MCF-7 human breast adenocarcinoma cells (Sophy et al. 2016). Furthermore, Araujo et al. (2019) noted that enzymatic treatment with cellulase and protease enhanced the antiproliferative effect of this plant extract against 786-O human renal carcinoma and PC-3 human prostate cancer cell lines.

### *Aloe vera* L. Burm.f.

The anticancer activity of the herbal plant *Aloe vera* was assessed against the human hepatocellular carcinoma cell line Hep-G2. The results revealed a dose-dependent repression of cancer cells at the IC_50_ value of 10.5 µg/ml. This plant showed cytotoxic activity by initiating Hep-G2 cell apoptosis through reduced B-cell lymphoma 2 (Bcl-2) and enhanced p53 expression (Shalabi et al. 2015). Further studies indicate that aloin and emodin are effective anticancer agents present in this plant. Aloin inhibits growth and angiogenesis in cancer cells, while emodin exhibits antiproliferative effects (Radha and Laxmipriya 2015).

### *Annona muricata* L.

*Annona muricata*, commonly known as Graviola, contains acetogenins in its seeds, leaves, and fruits, which block adenosine triphosphate synthesis and inhibit the transport of anticancer drugs outside the cell, thereby enhancing the effectiveness of chemotherapy. Previous studies indicate that acetogenins are effective against multidrug-resistant cancers. *In vitro* studies have revealed that the leaf extract of *A. muricata* exhibits antiproliferative effects on breast cancer cell lines, including malondialdehyde (MDA), SK-BR-3, and EACC (Gavamukulya et al. 2014) and the prostate cancer cell line BPH-1 by inducing apoptosis and reducing prostate size (Asare et al. 2015). The oral administration of an ethanolic extract of *A. muricata* to BALB/c mice implanted with 4T1 breast cancer cells demonstrated its anticancer efficacy, thus supporting its use in traditional Mexican medicine (Merlín-Lucas et al. 2021).

### *Annona squamosa* L.

The *Annona squamosa* plant belongs to the family *Annonaceae*; it is extensively used for treat microbial infections, inflammation, and cancer (Shehata et al. 2021). Recent studies indicate that extracts from its seeds, peel, and pulp show significant anticancer efficacy against diverse cancer cell lines such as MCF-7, HepG-2, Caco-2, and PC3 (Shehata et al. 2021). The seed extract of this plant demonstrated strong cytotoxicity and growth inhibition of various cancer cell lines, including asoral (KB), lung (A549), chronic myeloid leukemia (K-562), and MCF-7, without impairing normal lymphocytes (Vikas et al. 2019).

### *Arbutus andrachne* L.

*Arbutus andrachne* belongs to the *Ericaceae* family. Almost all parts of the *A. andrachne* plant, including roots, leaves, and fruits, have ethnomedical applications. The aerial parts of this plant exhibit strong anticancer effects on various cancer cell lines, including T-47D, MCF-7, HRT-18, Caco-2, A375.S2, and WM1361A. Substantial anticancer activity has been reported against MCF-7, A375.S2, and HRT-18 cell lines, whereas moderate cytotoxic effects were detected against Caco-2, T-47D, and WM1361A cell lines (Abu-rish et al. 2016).

### *Aristolochia ringens* Vahl

This plant, also known as *Howardia ringens*, is traditionally used for treating digestive issues, rheumatoid arthritis, piles, insomnia, and edema (Aigbe et al. 2019). The root extract of *A. ringens* exhibited anticancer activities against A431, A549, PC-3, HeLa, and the human acute monocytic leukemia cell line THP-1 (Akindele et al. 2015). *A. ringens* dose-dependently significantly inhibited tumor growth in S180 ascites and S180 solid tumor models. Moreover, it improved the average survival duration in *in vivo* models (L1210 lymphoid leukemia cells) (Daoudi et al. 2013).

### *Asplenium nidus* L.

*Asplenium nidus*, a medicinal fern, is a member of the *Aspleniaceae* family. A recent study extracted two bioactive flavonoids from *A. nidus*, including quercetin-7-O-rutinoside and gliricidin-7-O-hexoside. These compounds exhibited excellent anticancer effects against HeLa and Hep-G2 cells (Jarial et al. 2018).

### *Azadirachta indica A*. Juss.

*Azadirachta indica*, belonging to the *Meliaceae* family, is primarily distributed throughout the Indian subcontinent and contains several active compounds, including nimbin, nimbidol, nimbidin, and nimbolide. It has around 40 active limonoids compounds, which exhibit antiulcer, antimicrobial, antiviral, and anticancer properties (Guchhait et al. 2022). Takagi et al. (2014) purified 17 limonoid compounds and tested their activity against breast (SK-BR-3), lung (A549), stomach (AZ-521), and leukemia (HL-60) cancer cell lines; of these, 7 compounds showed significant cytotoxic activity. Nimonol exhibits cytotoxicity by inducing apoptosis (Takagi et al. 2014). Kitdamrongtham et al. (2014) isolated flavonoids from *A. indica* and reported that these compounds showed cytotoxicity against HL-60, SK-BR-3, AZ-521, and A549 cancer cell lines. Nimbolide inhibited cancer cell autophagy and promoted apoptosis through the phosphoinositide 3-kinase (PI3K)/Akt/glycogen synthase kinase-3 beta (GSK-3β) signaling pathway (Kitdamrongtham et al. 2014). Kashif et al. (2019) found that nimbolide-treated cancer cells (A549, PC-3, and DU-145) exhibited a significantly higher number of apoptotic and necrotic cells compared to untreated cells, with no apparent effects on normal cells (NIH/3T3 and CCD-18Co). Nimbolide-treated cancer cells showed high expression of caspases 3, 8, and 9. Additionally, ethanolic foliar extract suppresses the growth of cancer cells, particularly breast and cervical cancer cells, through apoptosis (Kashif et al. 2019).

### *Barleria grandiflora* Dalzell

*Barleria grandiflora* belongs to the *Acanthaceae* family. It shows cytotoxic effects on Dalton’s lymphoma ascites (DLA) and Vero cell lines. Manglani et al. (2014) demonstrated that the administration of *B. grandiflora* leaf extract to mice bearing DLA tumors reduced ascitic fluid and suppressed tumor growth, ultimately increasing the longevity of mice. A significant adverse effect associated with cancer chemotherapy often causes myelosuppression, leading to anemia. However, treatment with *B. grandiflora* leaf extract increased the hemoglobin level and red blood cell count and decreased white blood cell count, highlighting its anticancer potential (Manglani et al. 2014).

### *Berberis aristata* DC.

This shrub is also commonly known as Daru haldi or tree turmeric. The root extract of *Berberis aristata* showed cytotoxic activity toward A2780 (ovary), Hop-62 (lung), and DWD (oral) cancer cell lines (Gaidhani et al. 2013). Ethanol extract of *B. aristata* stem bark reduced weight gain due to tumor cell proliferation and increased the viability of Ehrlich ascites carcinoma (EAC)-bearing mice as compared to cisplatin reference (Pai et al. 2012). A recent study revealed that the root extract of this plant contains three important alkaloids, namely thalifendine, jatrorrhizine, and berberrubine, and exhibits significant antioxidant and antiproliferative effects on MCF-7. The docking analysis also confirmed that these alkaloids target the epidermal growth factor receptor (EGFR) of breast cancer cells (Kumari 2022).

### *Biancaea sappan* L. Tod

*Biancaea sappan*, commonly known as sappan wood or Indian redwood, is a flowering tree. Its ethyl acetate extract contains brazilin A compounds, which inhibit tumor cell growth by causing DNA strand scission (Bukke et al. 2018). Leaf and heartwood extracts of *B. sappan* were evaluated for significant anticancer efficacy against MCF-7 and A549 cells. Additionally, a docking analysis established that brazilin A acts by inhibiting the anti-apoptotic gene *BCL2* (Widodo et al. 2022).

### *Bidens pilosa* L.

*Bidens pilosa* is native to South America and is frequently used in ancient remedies worldwide, particularly in herbal teas for various ailments, including inflammation, diabetes, pharyngitis, enteritis, and bacillary dysentery. Its hydroalcoholic root preparations are utilized for treating malaria and tumors in the Amazon and other southern Brazilian regions (Singh et al. 2017a). *B. pilosa* extracts demonstrate several beneficial effects such as anti-inflammatory, immunosuppressive, chemopreventive, hepatoprotective, antimalarial, antimicrobial, and anticancer effects (Shen et al. 2018). Specific isolated compounds, for example, phenyl-1,3,5-heptatriyne, have been identified for their anticancer and antimalarial effects. Notably, petroleum ether extracts of this plant show cytotoxic activity against A549 cells (Shen et al. 2018). Furthermore, tris (2,4-di-tert-butylphenyl) derived from this plant extract exhibited promising cytotoxicity toward A549, HeLa, and Hep-G2 cell lines. *In vivo* confirmation of these findings was obtained using *Artemia salina, Caenorhabditis elegans*, and *Danio rerio* (Zahara et al. 2022). However, despite these observations, more research is required to fully understand the antitumor efficiency and active compounds of *B. pilosa*.

### *Calligonum comosum* L’Hér

*Calligonum comosum* is a leafless perennial shrub that produces several phytoconstituents, including phenolics, flavonoids, alkaloids, and essential oils. *C. comosum* extract showed cytotoxicity toward Hep-G2 cells at an IC_50_ value of 9.60 µg/ml (Shalabi et al. 2015). It contains major phytoconstituents such as kaempferol, catechin, and derivatives of these compounds, which enhance cytotoxicity by inducing apoptosis. It also decreases Bcl-2 protein expression by increasing *TP53* gene expression (Shalabi et al. 2015; Alehaideb et al. 2020).

### Carissa carandas

*Carissa carandas*, known as karonda in Asia, belongs to the *Apocynaceae* family and exhibits excellent antimicrobial, antioxidant, and anti-inflammatory potential (Saeed et al. 2024). Previous studies have indicated that its leaves as well as fully ripe and unripe fruits show significant cytotoxic effects against various cancers, including liver, lung, breast, cervical, bone, and ovarian cancers (Mishra et al. 2024). Bano et al. (2022) revealed three potent anticancer agents derived from the fresh leaves of this plant: uvaol, 23-hydroxy ursolic acid, and 27-O-Z-p-coumaroyl ursolic acid, which showed anticancer effects on NCI-H460 cancer cells. Additionally, carissic acid derived from *C. carandas* fruits showed potent anticancer acativity against HT-29 (colon), MCF-7, and A549 cancer cells by inducing apoptosis and inhibiting cell proliferation (Kour et al. 2024).

### *Cedrus deodara* (Roxb. ex D. Don) G. Don

*Cedrus deodara* is an evergreen coniferous tree known by its common name, Deodar cedar or Himalayan deodar or Himalayan cedar. Total lignans from *C. deodara* (CTL) needles were extracted and analyzed for anticancer activity against A549 cells. CTL exerted cytotoxic effects by inducing apoptosis (Shi et al. 2019). *C. deodara* bark extract also show cytotoxic effects on MCF-7 cells (Kumar et al. 2021). Furthermore, this plant extract demonstrated significant cytotoxicity against PC-3, A2780 (ovary), and Mia PaCa-2 cancer cell lines (Gaidhani et al. 2013).

### *Cenchrus ciliaris* L.

*Cenchrus ciliaris* is a herbaceous grass known by its common name, dhaman/anjan grass. Both aerial and root extract of *C. ciliaris* show cytotoxicity against Hep-G2, A549, MCF-7, and HeLa cells as well as against colon cell lines HCT-116 and Caco-2, with the highest efficacy toward Hep-G2 cells (Alothman et al. 2018).

### *Centaurea antiochia* Boiss

*Centaurea antiochia* is classified under the *Asteraceae* family (Bona 2013). Certain *Centaurea* species, such as *C. adjarica, C. bracteata, C. cataonica, C. cynaroides, C. dealbata, C. indurata, C. macrocephala, C. melitensis, C. nigrescens*, and *C. ruthenica*, have been documented to exhibit anti-inflammatory effects (Csupor et al. 2013). *C. antiochia* extract shows a dose-dependent anticancer effects on HeLa cells (Ozcan et al. 2016).

### *Cordia dichotoma* G. Forst

In India, *Cordia dichotoma* is used by various ethnic groups for its medicinal properties, including anticancer, astringent, antihelmintic, analgesic, antimicrobial, hepatoprotective, and diuretic properties (Raghuvanshi et al. 2022). Hussain et al. (2020) demonstrated the antitumor potential of *C. dichotoma* extract on EAC in Swiss albino mice (Hussain et al. 2020). Raina et al. (2022) reported its cytotoxic effects on several cancer cell lines, including SH-SY5Y, N2A, U-251, MCF-7, MDA-MB-231, A549, SW-620, HCT-116, MIA PaCa-2, and PANC-1, which originate from central nervous system, breast, lung, colon, and pancreas cancer cells; the authors reported that the extract contains compounds such as caffeic acid, cis-vaccenic acid, ferulic acid, gallic acid, palmitic acid, and vanillic acid. Usmani et al. (2018) found that the seed extract exhibited significant cytotoxicity against A549 and HeLa cells by inducing apoptosis in a dose-dependent manner from 25 to 250 µg/ml (Usmani et al. 2018).

### *Cotinus coggygria* Scop.

*Cotinus coggygria* (family *Anacardiaceae*) is found across southern Europe and the eastern region of Central Asia, stretching from the Himalayas to northern China. Indigenous populations use extracts of this plant owing to its antiseptic, anti-inflammatory, and hepatoprotective properties. This plant shows anticancer potential by inhibiting fibroblast activation protein α, with no adverse effects on normal human cell lines MCF-10A and BJ (Iliev et al. 2020). Flavonoids from *C. coggygria* inhibited cell proliferation with IC_50_ values of 93.57, 107.62, and 128.49 µg/ml against DBTRG-05MG, U251, and U87 glioblastoma cells, respectively. The anticancer effect was exerted through apoptosis induction by modulating the PI3K/Akt-extracellular signal-regulated kinase (ERK) cascade pathway. *In vivo* tests using xenograft animal models of glioblastoma revealed significant antitumor activity (Wang et al. 2015). Furthermore, at 48 h after treatment with the leaf extract, *HDAC5* and *HDAC7* mRNA transcripts were decreased in MCF-7 cells. Additionally, *HDAC3* gene expression was suppressed after 72 h of treatment (Gospodinova et al. 2017a); the extract exerted significant antiproliferative effects on MCF-7, T47D, HeLa, and ovarian (A2780) cancer cells, while sparing non-cancerous MCF-10 cells (Gospodinova et al. 2017b). Gospodinova et al. (2021) also observed that this plant extract induces S-phase cell cycle arrest and apoptosis; reduces colony formation; alleviates DNA damage; alters cellular thermodynamic properties; and decreases the expression of several transcripts such as *p300, MBD3, DNMT1*, and *DNMT3a*.

### *Croton caudatus* Geiseler

*Croton caudatus* is a scandent shrub widely utilized as an ethnomedicinal plant by the “Kuki” Indian tribe. A recent study revealed that the phytochemicals β-sitosterol, *(E)*-dodec-3-en-1-ol, and tricosan-1-ol, octacosanoic acid derived from the *C. caudatus* extract exhibited promising anticancer effects on A549 cells (Neipihoi et al. 2021). Another study demonstrated cytotoxic activity of the plant extract toward MCF-7, HeLa, and DL cell lines, with potential cytotoxicity specifically against DL cells (Rosangkima and Jagetia 2015).

### *Curcuma longa* L. and *Curcuma zedoaria* (Christm.) Roscoe

*Curcuma longa* and *C. zedoaria* belong to the *Zingiberaceae* family and offer significant health benefits. *C. longa* rhizome extract is extensively used in polyherbal medicine, particularly for cancer treatment. Furthermore, *C. zedoaria* rhizome extract demonstrated significant cytotoxicity toward DLA, EAC, and A549 cell lines through apoptosis induction (Shehna et al. 2022). Curcumin, a polyphenolic compound from *C. longa* rhizome, shows strong anticancer effects against Hep-G2 cells at 1000 µg/ml concentration (Srivastava and Srivastava 2015); it also inhibits glioblastoma cell proliferation by altering various pathways and promoting the expression of several tumor suppressor proteins, including p16, p21, p53, caspase-3, caspase-8, caspase-9, Egr-1, c-Jun N-terminal kinase, Elk-1, ERK, and Bax. Additionally, curcumin reduces the expression of the proteins Bcl-2, Bcl-xL, mechanistic target of rapamycin (mTOR), p65, nuclear factor-kappa B (NF-κB), EGFR, retinoblastoma protein, cyclin D1, cell division cycle protein 2, cell myelocytomatosis oncogenes, and Akt (Vallianou et al. 2015). Curcumin also suppresses breast cancer growth by reducing NF-κB expression and potentially affecting the EGFR signaling pathway (Starok et al. 2015).

### Cyclea peltata

*Cyclea peltata* is a plant belonging to the *Menispermaceae* family and is commonly found across south and east India. *C. peltata* root extract is widely utilized in traditional remedies to treat certain health conditions such as jaundice, stomach pain, and smallpox. The root of this plant contains various bioactive compounds, primarily alkaloids, including fangchinoline and several forms of tetrandrine. Tetrandrine is known for its antioxidant, anti-inflammatory, antifibrotic, immunosuppressive, plasma glucose lowering, free radical scavenging, and anticancer properties (Shine et al. 2014). A recent study reported that tetrandrine derived from this plant exerts prominent cytotoxic effects on MDA-MB-231 and PANC-1 cancer cell lines by activating programmed cell death through reactive oxygen species generation (Chandrashekar et al. 2019).

### *Delphinium staphisagria* L.

*Delphinium staphisagria* (Entele grass) belongs to the *Ranunculaceae* family; it reaches a height of 1.5 m and contains essential oils and alkaloids (Bousfiha et al. 2016). Akgun et al. (2022) assessed the cytotoxic effects of this plant extracts on A549, MCF-7, HT-29, and HeLa cells in a dose- and time-dependent manner. The authors also assessed the cellular toxicity of these extracts on healthy BEAS 2B cells. Moreover, cell death mechanisms, through apoptosis or necrosis, were evaluated using markers such as activated caspase 3, caspase-cleaved cytokeratin-18, and cleaved poly(ADP-ribose) polymerase by fluorescent imaging (Akgun et al. 2022).

### *Dillenia pentagyna* Roxb.

Screening of the ethanolic leaf extract of *Dillenia pentagyna* revealed its cytotoxic efficacy against U2OS, A549, and HeLa cells, with A549 cells exhibiting higher sensitivity toward the treatment. The mechanism of action involves the induction of the fundamental pathways of apoptosis (De et al. 2023). The bark and stem extract also showed cytotoxicity toward DL, MCF-7, and HeLa cells at concentrations of 25.8, 41.6, and 76.8 µg/ml, respectively. Animals treated with *D. petnagyna* extract exhibited a significant reduction in glutathione level, which is directly associated with tumor proliferation initiation through protein kinase C regulation. Therefore, *D. petnagyna* has been proven to be beneficial for cancer treatment as it induces glutathione depletion (Rosangkima et al. 2008).

### Eclipta alba

*Eclipta alba*, also known as Bhringaraj in Ayurveda, belongs to the *Asteraceae* family and is recognized for its beneficial effects on liver and hair. Its well-documented medicinal properties include antimycotoxic, antibacterial, antioxidant, antihepatotoxic, anticancer, and rejuvenating effects. *E. alba* contains compounds such as coumestan, wedelolactone, and β-sitosterol, which contribute to liver protection and antioxidant and anticancer effects (Nelson et al. 2020; Arya et al. 2015). Furthermore, *E. alba* extracts exhibit dose-dependent anticancer activity against various cancer cell lines, including MCF-7, HCT-116, PC-3, and the renal cell carcinoma cell line RCC-45, with notably high efficacy observed in HCT-116 cells, without adverse effects on normal embryonic lung fibroblast WI-38 cells (Nelson et al. 2020; Arya et al. 2015). Significant anticancer effects were also observed against MDA-MB-231 and MCF-7 cells as well as in a 4T1 syngeneic mouse model through the activation of mitochondrial apopto tic pathways and significant upregulation Hsp60 local ization (Arya et al. 2015). Recent studies have identified bioactive compounds in *E. alba*, including ecliptalbine, wedelolactone, ursolic acid, and β-amyrin. These compounds were evaluated with an *in silico* approach and showed a strong affinity for breast cancer target proteins such as matrix metalloproteinase, estrogen receptor, and progesterone receptor. This observation confirms their excellent antioxidant and anticancer efficacy against MDA-MB-231 cells (Mani et al. 2024).

### *Euphorbia hirta* L.

*Euphorbia hirta*, also known as Dugdhika in Ayurveda, shows antimicrobial, antimalarial, anti-inflammatory, antioxidant, and antidiarrheal effects and is often utilized for treating gastrointestinal disorders, respiratory diseases, and conjunctivitis. The *E. hirta* plant includes various bioactive compounds such as β-amyrin; β-sitosterol; euphorbin A, B, C, and D; gallic acid; heptacosane; kaempferol; n-nonacosane; protocatechuic acid; quercetin; rutin; 1,3,4,6-tetra-O-galloyl-β-D-glucose; 2,4,6-tri-O-galloyl-β-D-glucose; and 24-methylene cycloartenol (Kumar et al. 2010). *E. hirta* plant extract shows promising antitumor effects on Hep-G2 cells by inhibiting cell proliferation and triggering apoptosis (Kalaivani et al. 2024). Another study investigated its dose-dependent anticancer effects using the EAC model in mice. The findings revealed that treatment with the *E. hirta* plant extract reduced both tumor volume and weight and increased lifespan when administered at a dose of 200 mg/kg (Sulaiman et al. 2023).

### Euphorbia tirucalli L.

*Euphorbia tirucalli* is a small tree whose latex contains bi- and tri-terpenoid, which exhibit anticancer activity. The leaf and stem extracts of this plant show cytotoxic effects on Mia PaCa-2 cells (pancreas cancer cell line) (Munro et al. 2015). Latex of *E. tirucalli* primarily contains the triterpenes euphol and tirucallol, which are mainly responsible for anticancer activity (De Souza et al. 2019). In another study, euphol exhibited significant anticancer effects on 73 cancer cell lines, originating from 15 different human tumor types, with IC_50_ values ranging from 1.4 to138.89 µM (Silva et al. 2018).

### *Ficus beecheyana* Hook. & Am.

*Ficus beecheyana* is a member of the *Moraceae* family. Its root extract inhibited the survival of HL-60 cells by triggering apoptosis. Moreover, it also increased the expression levels of Bad, Bak, and Bax while decreasing Bcl-2 protein levels. It exerts cytotoxic effects through Fas and mitochondrial-dependent pathways. Several bioactive phytochemicals, including caffeic acid, gallic acid, and rutin, are mainly responsible for this effect (Yen et al. 2018).

### *Ficus carica* L.

*Ficus carica* is a small deciduous tree with notable cytotoxic properties. Its leaf latex showed cytotoxicity toward MDA-MB-231 cells by reducing the expression of several proteins, including adenosine monophosphateactivated protein kinase alpha, cAMP response element-binding protein, ERK, and GSK-3α/β (Al Ghalban et al. 2021). Acetone-based leaf extract of *F. carica* exhibited promising cytotoxicity against Hep-G2 cells at 0.179 mg/ml concentration, while its latex containing ficin showed efficacy against HT-29 and PC-3 cancer cells (Mustafa et al. 2021). Additionally, *F. carica* leaf extract increased the expression of apoptosis-promoting genes and reduced cell mobility in MDA-MB-231 cells (Zhang et al. 2018).

### Garcinia indica

*Garcinia indica* (family *Clusiaceae*), also known as kokum, is native to India and widely distributed in Africa, Asia, and western Polynesia. This plant is extensively utilized to treat dermatitis and diarrhea and improve digestion. *G. indica* contains several important bioactive compounds, including cyanidin-3-glucoside, cyanidin-3-sambubioside, garcinol, and hydroxy-citric acid (Lim et al. 2021). Garcinol derived from the fruit peel and leaves of *G. indica* exhibits remarkable anticancer properties against esophageal, gastric, colorectal, pancreatic, and liver cancers (Lim et al. 2021). Recent studies suggest that garcinol inhibits histone acetyltransferases and disrupts the regulation of microRNAs (miRNAs), which are primarily responsible for tumor expansion (Kopytko et al. 2021). Previous studies have also shown that garcinol inhibits cell growth and triggers programmed cell death in different cancer cells, particularly in DU145, MDA-MB-231, and BxPC-3 cancer cells. The efficacy of garcinol in reducing tumor growth was confirmed through *in vivo* assays using the MDAMB-231 xenograft mice model (Ahmad et al. 2012).

### *Helicteres isora* L.

*Helicteres isora* is a shrub known by its vernacular name, East Indian screw tree. The acetone-based fruit extract of this plant showed cytotoxicity toward NCIH460 cancer cells (Raaman and Balasbramanian 2012). The entire plant has been screened for cytotoxic activity against Hep-3B, HL-60, PN-15, and HeLa-B75 cells, and a moderate anticancer effect on these cell lines was observed. The major cytotoxic compounds of this plant are isocucurbitacin B and cucurbitacin B (Shaikh et al. 2014). Fruit extract of *H. isora* showed differentiation-inducing properties in the colon cancer cell line RCM-1 (Rattanamaneerusmee et al. 2018).

### *Hibiscus rosa-sinensis* L.

This is a small bushy shrub containing several bioactive molecules, including phenolics, flavonoids, anthocyanins, and organic acids. Plant extracts of *Hibiscus rosa-sinesis* are reported to show anticarcinogenic effects because of their antioxidant and hypolipidemic properties (Al-Saily et al. 2018; Rasul et al. 2023). A phenolic extract of *H. rosa-sinensis* showed cytotoxicity against a macrophage cancer cell line (RAW 264.7) and a testicular cell line (Tera-1). Tera 1 cells showed more sensitivity at an IC_50_ value of 23.496 mg/ml compared to RAW 264.7 cells (IC_50_ value: 31.841 mg/ml) (Al-Saily et al. 2018). A recent study investigated potent anticancer phytochemicals in *H. rosa-sinensis* flowers, specifically targeting the mammalian target of rapamycin (mTOR) through docking analysis. The results showed that stigmastadienol, lupeol, and taraxasterol acetate exhibited strong binding affinities to mTOR compared to the control group. Thus, these compounds could serve as effective mTOR inhibitors for breast cancer therapy (Rasul et al. 2023).

### Hibiscus sabdariffa

In Ayurveda, the plant *Hibiscus sabdariffa* (family *Malvaceae*) has been utilized to treat conditions such as hypertension, hypercholesterolemia, hyperlipidemia, diarrhea, and other ailments. Recently, its anticancer activities as well as antiproliferative, antimutagenic, apoptotic, antiangiogenic, and antimetastatic properties have been widely investigated in both human cancer cells and animal studies (Laskar and Mazumder 2020). Moreover, the *in vivo* anticancer effects of the extract from *H. sabdariffa* calyxes was evaluated using Wistar rats. The results demonstrated that the extract significantly decreased tumor volume by increasing superoxide dismutase activity and decreasing MDA levels (Bassong et al. 2022).

### *Hymenodictyon excelsum* (Roxb.) Wall.

*Hymenodictyon excelsum*, belonging to the *Rubiaceae* family, has been studied for its cytotoxic and apoptotic activities. The methanolic extract from *H. excelsum* bark demonstrated cytotoxic activity against L-929 cells in a 3-(4,5-dimethylthiazol-2-yl)-2,5-diphenyltetrazolium bromide (MTT) assay and significantly inhibited the growth of DLA cells in a trypan blue dye exclusion assay. The apoptotic and cytotoxic effects are attributable to morphological changes such as cell shrinkage, chromatin condensation, and nuclear membrane blebbing (Khairunnisa and Karthik 2014). Studies have also demonstrated the anticancer potential of *H. excelsum* against HT-29 and Hep-G2 cells based on the presence of anthraquinone and coumarin compounds (Chakraborty et al. 2017). A molecular docking study identified several phytochemicals, including damnacanthal, esculin, lucidin, morindone, and soranjidiol, with promising binding affinity to the androgen receptor, similar to dihydrotestosterone, highlighting their potential for further research (Rahman 2015).

### *Hypericum kotschyanum* Boiss.

The genus *Hypericum* is known worldwide for its medicinal benefits. Aerial parts of this plant showed moderate cytotoxicity toward HeLa and Vero cell lines at 507 and 367 µg/ml concentrations, respectively (Artun et al. 2016).

### *Inula viscosa* L. Aiton

*Inula viscosa*, a therapeutic herb from the *Compositae* family, shows anticancer activity against human malignant melanoma cells, particularly A2058 and MeWo. Its methanolic extract induces cell death in these cells in a dose- and time-dependent manner by substantially altering miRNA expression. It downregulates oncogenic miR-191 and miR-193 and upregulates onco-suppressive miR-524 and miR-579 (Colak et al. 2022). Additionally, it repressed the development of 7,12-dimethylbenz(a) anthracene-induced skin carcinoma in mice (El Yaagoubi et al. 2021). Tomentosin, a phytochemical derived from this plant, exhibits antiproliferative effects on human Burkitt’s lymphoma cancer cells by activating apoptosis and suppressing the cell cycle process. It inhibits growth-related genes such as *JAK/STAT* and *PI3K/AKT*, downregulates the antiapoptotic gene *CDKN1A* and *BCL2A1*, and upregulates the proapoptotic gene *PMAIP1* (Virdis et al. 2021). Tomentosin also exhibits cytotoxicity towards MOLT-4 cells by preventing NF-κB activation through the mTOR/PI3K/AKT pathway (Yang et al. 2021).

### *Lagenaria siceraria* (Molina) Standl.

This plant, classified under the *Cucurbitaceae* family, shows a wide range of medicinal benefits, including antioxidant, antibacterial, antituberculosis, antihelmintic, antidiarrheal, antidiabetic, antihypertensive, analgesic, laxative, hepatoprotective, cardioprotective, and immunosuppressive effects. According to a recent study, *L. siceraria* fruit extract exhibits promising dose-dependent cytotoxicity toward HT-29 and MCF-7 cells (Attar and Ghane 2019). Cucurbitacin I, a crucial component of this plant, inhibits tumor growth by affecting apoptosis and targeting tumor blood vessel formation (Attar and Ghane 2018; Attar and Ghane 2019).

### *Lannea coromandelica* (Houtt.) Merr.

*Lannea coromandelica*, also known as the Indian ash tree, grows in south and southeast Asia. The bark and foliage extracts of this plant are highly cytotoxic. An *in vivo* study examined the influence of chloroformbased bark extract on mice seeded with EAC. After 7 days of treatment, the life span of treated mice increased by 33.3% (Hossain et al. 2018). Another study reported that the ethanolic extracts of twigs from *L. coromandelica* induced apoptosis in Hep-G2 cells. A gas chromatography–mass spectrometry analysis revealed that 2-palmitoylglycerol, hexadecanoic acid, and octadecenoic acid are the primary bioactive components in crude extracts (Weerapreeyakul et al. 2016). However, there are fewer positive reports describing the presence of anticancer components in *L. coromandelica*, indicating a significant scope for further research on potential pharmaceutical compounds in this species.

### *Leea indica* (Burm.f.) Merr

*Leea indica*, belonging to the *Leeaceae* family, originates from the tropical areas of India, Bangladesh, China, Bhutan, and Malaysia. The leaf of *L. indica* provides several therapeutic benefits in treating diabetes, diarrhea, and dysentery (Ghagane et al. 2017; Neo et al. 2023). The plant-derived leaf extract exhibited excellent anticancer efficacy against DU-145 and PC-3 cells (Ghagane et al. 2017). Another study demonstrated the significant cytotoxic properties of this plant extract (leaf) and its phytochemical methyl gallate when combined with oxaliplatin and natural killer (NK) cells. The findings suggest that ovarian cancer cells, including SK-OV-3 and OVCAR-5, became more susceptible to NK cells. Additionally, the plant extract reduced the production of proinflammatory cytokines TNF-α and interleukin-1 beta (Neo et al. 2023).

### *Moullava digyna* (Rottler) Gagnon & G.P.Lewis

This plant, generally named Vakul Mul, is categorized in the *Leguminosae* family. It contains homoisoflavonoids, commonly present in many fruits, beverages, vegetables, etc. Intricatinol, isolated from this plant, shows anticancer effects on A549 lung cancer cells. This compound enhances caspase-dependent apoptosis in A-549 cells when combined with a wellknown anticancer drug, i.e., cisplatin. Furthermore, intricatinol activates the p38 mitogen-initiated protein kinases/p53 signaling pathway and damages DNA in A549 cells (Singh et al. 2017b).

### *Myristica fragrans* Houtt.

*Myristica fragrans* is an aromatic tree, commonly known as the nutmeg plant, and is recognized for its spicy products and essential oil, which is mostly used in fragrance, flavor, and pharmaceutical industries. This plant is native to Indonesia and primarily occurs in tropical regions and coastal areas (Ibrahim et al. 2020). Rengasamy et al. (2018) assessed the anticancer efficacy of this plant extract by the MTT assay against KB cell lines and demonstrated its ability to inhibit cancer cell growth. *M. fragrans* contains the bioactive compound myristicin, which is responsible for its significant cytotoxic effect (Rengasamy et al. 2018). Ginting et al. (2021) also identified anticancer properties of the root extract of *M. fragrans* against MCF-7 cells and discovered a lignan compound (6′-methyl-(7-hydroxy-8-methylbut-9-en)-3,2′-dimethoxybiphenyl-4,5-diol)) as an antitumor agent.

### *Ocimum sanctum* L.

*Ocimum sanctum*, also known by its common name “Tulsi,” is the holy herb of India and belongs to the *Lamiaceae* family. *O. sanctum* exhibits several therapeutic properties and has been widely utilized in medical practice since ancient times. Essential oil from *O. sanctum* leaves demonstrated dose-dependent anticancer effects on MCF-7 cells by inducing apoptosis at an IC_50_ value of 170 µg/ml. Moreover, its leaf extracts increase the gene expression of *Bid, Bax, Bcl-2*, and *p53*, thereby confirming apoptosis induction (Manaharan et al. 2016). Another study revealed that the plant extract promotes apoptosis and suppresses PC-3 cell proliferation *in vitro* by downregulating genes such as *p65, FAK*, and activated *ERK-1/2*, while its intraperitoneal administration significantly hinders tumor growth *in vivo* (Shimizu et al. 2013). *O. sanctum* contains vicenin-2, which induces G2/M phase cell cycle arrest in HT-29 cells, triggers apoptosis, increases proapoptotic gene expression, and decreases *BCL2* gene transcription (Yang et al. 2018).

### *Opuntia ficus-indica* L. Mill

This plant is classified into the *Cactaceae* family. Its various components, including roots, stems, cladodes, fruits, peels, and seeds, were shown to exert anticancer effects in cellular experiments. Multiple studies reported the presence of various medicinally important compounds in this plant, including betanin, catechin, gallic acid, naringenin, naringin, protocatechuic acid, quercetin, and quinic acid (Wang et al. 2023). Another study emphasized the influence of the components of this plant on the anti-oncogene p16INK4a through the demethylation of its promoter and increased gene expression (Naselli et al. 2014).

### *Phyllanthus emblica* L.

*Phyllanthus emblica*, or Indian gooseberry, is a medicinal plant renowned for the widespread utilization of its fruit extract in traditional remedies for liver issues, constipation, diabetes, anemia, and tumors (Liu et al. 2012; Liu et al. 2024; Chekdaengphanao et al. 2022). Recent studies have highlighted the immuneenhancing and anticancer effects of various compounds derived from *P. emblica*, including geraniin, glucopyranoside, isocorilagin, kaempferol, kaempferol 3-β-d-glucopyranoside, quercetin, and rutin, on human embryonic lung fibroblast (HELF) and MCF-7 cells. The results of this study revealed that isocorilagin and geraniin exhibited higher cytotoxicity toward MCF-7 cells compared to the other compounds, influencing both cytotoxicity and inhibition of splenocyte proliferation in HELF and MCF-7 cells (Liu et al. 2012). Myricetin has also been reported to target carbonic anhydrase II in cancer pathways (Liu et al. 2024). Additionally, *P. emblica* herbal extract emonstrated anticancer effects on cholangiocarcinoma cell lines by promoting apoptosis and regulating the expression of key proteins caspase-3, caspase-9, Bax, and p53, while reducing Bcl-2 transcription (Chekdaengphanao et al. 2022). The plant has also shown potential against human papillomavirus-associated cervical cancers by suppressing the expression of oncogenes linked to cancer progression (Mahata et al. 2013).

The cytotoxic properties of *Piper betle, Portulaca oleracea* L., *Ricinus communis* L., *Saraca asoca* (Roxb.) Willd., *Saurauia roxburghii* Wall., *Tabernaemontana divaricata* (L.) R.Br. ex Roem. & Schult., *Teucrium polium* L., *Thymus vulgaris* carvacrol L., *Tillandsia recurvata* L., *Tribulus terrestris, Vitis vinifera* L., *Withania coagulans* (Stocks) Dunal, *Withania somnifera* L. Dunal, *Zea mays* L., and *Zingiber officinale* Roscoe are described in Supplementary Table 2.

### Recent advances and opportunities in the discovery of novel anticancer drugs

Recent advancements in analytical chemistry, bioinformatics tools, and computational analysis have significantly improved our capability to investigate plant species that have been traditionally used across different cultures worldwide. By precisely characterizing their chemical compositions and evaluating their therapeutic activities, researchers are a step closer to establish important relationships between the two (Zhang et al. 2017). In recent years, there has been a global trend to create digital databases and resources that systematically catalog the active ingredients found in various herbs and herbal preparations, together with pertinent data (Ingle et al. 2024). In this context, Indian scientists have made significant achievements in creating digital databases for plant-based medicinal properties, including Phytochemica (Pathania et al. 2015), NeMed Plant (Meetei et al. 2012), MedPServer (Potshangbam et al. 2019), IMPPAT 2.0 (Vivek-Ananth et al. 2023), SuperNatural 3.0 (Gallo et al. 2023), and HIT 2.0 (Yan et al. 2022). However, many studies tend to address only the superficial aspect of the vast diversity of medicinal plants Repositories such as CancerResource and NPACT link cancerrelated compounds to their targets, NPACT in particular, highlighting the anticancer activities of phytochemicals derived from *in vitro* and *in vivo* research studies (Ahmed et al. 2011; Mangal et al. 2013). These repositories serve as a crucial resource underscoring the significant potential of Indian herbs in the realm of future drug discovery. However, it is important to clearly differentiate between pharmacological properties observed in wet laboratories and those predicted through *in silico* methods.

Advancements in molecular modeling techniques, such as molecular docking and molecular dynamics, play a crucial role in preclinical drug discovery. These techniques are essential for understanding how phytochemical analogs interact with their target proteins (Ferreira et al. 2015). Successful applications of computeraided drug design are exemplified by several clinically approved drugs such as imatinib, dasatinib, and ponatinib. Nevertheless, these techniques encounter several challenges owing to the unavailability of three-dimensional structures of lesser-known anticancer compounds (Yi et al. 2018). Recent years have witnessed significant advancements in phytochemical extraction methods to address the environmental concerns associated with harvesting large quantities of plant materials (Liu et al. 2016). Therefore, the biosynthesis of natural medicines has garnered considerable interest, exemplified by success stories for artemisinin, paclitaxel, tanshinone, breviscapine, noscapine, and thebaine (Li et al. 2021). Unfortunately, almost 40% of natural bioactive components cannot be chemically synthesized (Potshangbam et al. 2019). Recent advances in biotechnology and metabolic engineering, particularly through heterologous expression systems and the use of elicitors in plant tissue culture, have facilitated increased production of anticancer compounds (Singh et al. 2020). In this regard, Indian research groups have worked diligently to improve extraction methods for anticancer phytochemicals such as camptothecin and tetrandrine (Srivastava et al. 2005). For example, *C. acuminata* Decaisne is a primary source of camptothecin worldwide; however, overharvesting of this plant has endangered its survival in many countries, particularly in China. As a solution, Indian researchers have shifted their focus to the plant *Nothapodytes foetida* for extracting camptothecin. Unfortunately, this plant produces camptothecin in considerably smaller quantities. Therefore, to improve the yield, a new method was developed to isolate camptothecin from the twigs and stems of *N. foetida*, which achieved a 36% increase in yield compared to that using previous methods (Srivastava et al. 2005). Thus, it is crucial to conduct more research on the diverse plant species from the Indian subcontinent to obtain specific phytochemicals and conserve endangered plants.

Recent studies have demonstrated that various phytochemicals show promising anticancer properties without damaging normal cells. However, their poor absorption and low bioavailability restrict their medicinal use. Therefore, strategies such as liposomal formulations, nanoformulations, and development of structural analogs of these phytochemicals are highly effective to overcome these issues (Lagoa et al. 2020). Consequently, more research in this direction is needed to improve the understanding of their bioavailability, bioactivity, and metabolism.

## Conclusions

Medicinal plants indigenous to the Indian subcontinent have a key role in the advancement of novel pharmacological anticancer therapies. The overall progress in this field suggests that discovering new plant-derived anticancer drugs requires comprehensive *in silico* virtual screenings, preclinical studies, and pharmacokinetic evaluations of lesser-known plants and their phytochemicals in the Indian subcontinent. These strategies can enhance our understanding of novel plant-based anticancer medications and facilitate their discovery and development. Nonetheless, accurately predicting the roles of lesser-known phytochemicals in addition to traditional active compounds remains a significant challenge. Most studies often show methodological inadequacies, including the absence of control groups, small sample sizes, and short trial durations, which demand comprehensive clinical trials to assess toxicity and therapeutic potential. Combining modern advanced techniques and traditional medicines could enhance the introduction of novel phytochemicals into the market.

## Supplementary Material



## References

[cit0001] Abu-rish EY, Kasabri V, Hudaib MM, Mashalla SH, Al-Alawi LH, Tawaha K, Mohammad MK, Mohamed YS, Bustan-ji Y. 2016. Evaluation of antiproliferative activity of some traditional anticancer herbal remedies from Jordan. Trop J Pharm Res. 15: 469–474.

[cit0002] Ahmad A, Sarkar SH, Aboukameel A, Ali S, Biersack B, Seibt S, Li Y, Bao B, Kong D, Banerjee S, Schobert R. 2012. Anticancer action of garcinol *in vitro* and *in vivo* is in part mediated through inhibition of STAT-3 signaling. Carcinogenesis. 33(12): 2450–2456. 10.1093/carcin/bgs290 22971573

[cit0003] Ahmed J, Meinel T, Dunkel M, Murgueitio MS, Adams R, Blasse C, Eckert A, Preissner S, Preissner R. 2011. CancerResource: a comprehensive database of cancerrelevant proteins and compound interactions supported by experimental knowledge. Nucleic Acids Res. 39(Database issue): D960–D967. 10.3390/10.1093/nar/gkq910. PMC3013779 20952398

[cit0004] Aigbe FR, Sofidiya OM, James AB, Sowemimo AA, Akindere OK, Aliu MO, Dosunmu AA, Chijioke MC, Adeyemi OO. 2019. Evaluation of the toxicity potential of acute and subacute exposure to the aqueous root extract of *Aristolochia ringens* Vahl. (*Aristolochiaceae*). J Ethnopharmacol. 244: 112150. 10.1016/j.jep.2019.112150. 31401320

[cit0005] Akgun O, Akgun H, Sahin C, Celikler S, Ari F. 2022. *Angelica sylvestris* and *Delphinium staphisagria* extracts induce antiproliferation through caspase-mediated apoptosis on human cancer cells. Braz Arch Biol Technol. 65: e22210065. 10.1590/1678-4324-2022210065.

[cit0006] Akindele AJ, Wani Z, Mahajan G, Sharma S, Aigbe FR, Satti N, Adeyemi OO, Mondhe DM. 2015. Anticancer activity of *Aristolochia ringens* Vahl. (*Aristolochiaceae*). J Tradit Complement Med. 5: 35–41. 10.1016/j.jtcme.2014.05.001 PMC4488040 26151007

[cit0007] Al Ghalban FM, Khan AA, Khattak MNK. 2021. Comparative anticancer activities of *Ficus carica* and *Ficus salicifolia* latex in MDA-MB-231 cells. Saudi J Biol Sci. 28: 3225–3234. 10.1016/j.sjbs.2021.02.061 PMC8176001 34121859

[cit0008] Alehaideb Z, AlGhamdi S, Yahya WB, Al-Eidi H, Alharbi M, Alaujan M, Albaz A, Tukruni M, Nehdi A, Abdulla MH, et al. 2020. Anti-proliferative and pro-apoptotic effects of *Calligonum comosum* (L’Hér.) methanolic extract in human triple-negative MDA-MB-231 breast cancer cells. J Evid Based Integr Med. 25: 2515690X20978391. 10.1177/2515690X20978391. PMC7734547 33302699

[cit0009] Alothman EA, Awaad AS, Al-Qurayn NA, Al-Kanhal HF, El-Meligy RM, Zain YM, Alasmary FA, Alqasoumi SI. 2018. Anticancer effect of *Cenchrus ciliaris L*. Saudi Pharm J. 26: 952–955. 10.1016/j.jsps.2018.05.003 PMC6218845 30416351

[cit0010] Al-Saily HM, Al-Halbosiy MM, Al-Hady FN. 2018. Cytotoxic and apoptotic effects of phenolic extract of *Hibiscus rosasinensis* Linn flowers against cancer cells and normal cells. Indian J Public Health Res Dev. 9: 188–192.

[cit0011] Anand U, Dey A, Chandel AKS, Sanyal R, Mishra A, Pandey DK, De Falco V, Upadhyay A, Kandimalla R, Chaudhary A, et al. 2023. Cancer chemotherapy and beyond: current status, drug candidates, associated risks and progress in targeted therapeutics. Genes Dis. 10: 1367–1401. 10.1016/j.gendis.2022.02.007 PMC10310991 37397557

[cit0012] Araujo NMP, Pereira GA, Arruda HS, Prado LG, Ruiz ALTG, Eberlin MN, de Castro RJS, Pastore GM. 2019. Enzymatic treatment improves the antioxidant and antiproliferative activities of *Adenanthera pavonina* L. seeds. Biocatal Agric Biotechnol. 18: 101002. 10.1016/j.bcab.2019.01.040.

[cit0013] Artun T, Karagoz A, Ozcan G, Melikoglu G, Anil S, Kultur S, Sutlupinar N. 2016. *In vitro* anticancer and cytotoxic activities of some plant extracts on HeLa and Vero cell lines. J BUON. 21: 720–725. 27569095

[cit0014] Arya RK, Singh A, Yadav NK, Cheruvu SH, Hossain Z, Meena S, Maheshwari S, Singh AK, Shahab U, Sharma C, et al. 2015. Anti-breast tumor activity of Eclipta extract *in-vitro* and *in-vivo*: novel evidence of endoplasmic reticulum specific localization of Hsp60 during apoptosis. Sci Rep. 5: 18457. 10.1038/srep18457. PMC4682077 26672742

[cit0015] Asare GA, Afriyie D, Ngala RA, Abutiate H, Doku D, Mahmood SA, Rahman H. 2015. Antiproliferative activity of aqueous leaf extract of *Annona muricata* L. on the prostate, BPH-1 cells, and some target genes. Integr Cancer Ther. 14: 65–74. 10.1177/1534735414550198 25411208

[cit0016] Attar UA, Ghane SG. 2018. Optimized extraction of anti-cancer compound–cucurbitacin I and LC–MS identification of major metabolites from wild bottle gourd (*Lagenaria siceraria* (Molina) Standl.). S Afr J Bot. 119: 181–187.

[cit0017] Attar UA, Ghane SG. 2019. *In vitro* antioxidant, antidiabetic, antiacetylcholine esterase, anticancer activities and RP-HPLC analysis of phenolics from the wild bottle gourd (Lagenaria siceraria (Molina) Standl.). S Afr J Bot. 125: 360–370.

[cit0018] Banerjee P, Castellanos MR, Debata PR, Szerszen A, Research Foundation of City University of New York, Feinstein Institute for Medical Research. 2016. Activity enhancing curcumin compositions and methods of use. U.S. Patent Application 15/038,385.

[cit0019] Bano Z, Begum S, Ali SS, Kiran Z, Siddiqui BS, Ahmed A, Khawaja S, Fatima F, Jabeen A. 2022. Phytochemicals from *Carissa carandas* with potent cytotoxic and anti-inflammatory activities. Nat Prod Res. 36: 1587–1592. 10.1080/14786419.2021.1886101 33583281

[cit0020] Bassong TR, Kenmogne LV, Awounfack CF, Ndinteh DT, Njamen D, Zingue S. 2022. Effects of *Hibiscus sabdariffa* calyxes aqueous extract on antioxidant status and histopathology in mammary tumor-induced in rats. Evid Based Complement Alternat Med. 2022: 9872788. 10.1155/2022/9872788. PMC9056213 35502172

[cit0021] Bona M. 2013. An overview to *Centaurea (Asteraceae)* based on herbarium specimens of ISTE. J Fac Pharm İstanbul Univ. 43: 121–137.

[cit0022] Bousfiha A, Mejrhit N, Azdad O, El Kabbaoui M, Chda A, Tazi A, Bencheikh R, Aarab L. 2016. Immunomodulating properties of protein fractions isolated from *Delphinium staphisagria* seeds. J Med Plant Res. 10: 29–34.

[cit0023] Bukke AN, Hadi FN, Babu KS. 2018. *In vitro* studies data on anticancer activity of *Caesalpinia sappan L*. heartwood and leaf extracts on MCF7 and A549 cell lines. Data Brief. 19: 868–877. 10.1016/j.dib.2018.05.050 PMC5997910 29900385

[cit0024] Cavalier H, Trasande L, Porta M. 2023. Exposures to pesticides and risk of cancer: evaluation of recent epidemiological evidence in humans and paths forward. Int J Cancer. 152: 879–912. 10.1002/ijc.34300 PMC9880902 36134639

[cit0025] Chakraborty P, Sasi S, Nair AA, Anjum N, Tripathi YC. 2017. Medicinal applications, phytochemistry and pharmacology of *Hymenodictyon excelsum* (Roxb.) Wall: a review. Org Med Chem Int J. 2: 60–64.

[cit0026] Chandra S, Gahlot M, Choudhary AN, Palai S, de Almeida RS, de Vasconcelos JEL, dos Santos FAV, de Farias PAM, Coutinho HDM. 2023. Scientific evidences of anticancer potential of medicinal plants. Food Chem Adv. 2: 100239. 10.1016/j.focha.2023.100239.

[cit0027] Chandrashekar KR, Prabhu A, Rekha PD. 2019. Tetrandrine isolated from *Cyclea peltata* induces cytotoxicity and apoptosis through ROS and caspase pathways in breast and pancreatic cancer cells. In Vitro Cell Dev Biol Anim. 55: 331–340. 10.1007/s11626-019-00332-9 30945115

[cit0028] Chauhan R, D’Souza HL, Shabnam RS, Abraham J. 2015. Phytochemical and cytotoxicity analysis of seeds and leaves of *Adenanthera pavonina*. Res J Pharm Technol. 8: 198–203.

[cit0029] Chekdaengphanao P, Jaiseri D, Sriraj P, Aukkanimart R, Prathumtet J, Udonsan P, Boonmars T. 2022. Anticancer activity of *Terminalia chebula, Terminalia bellirica*, and *Phyllanthus emblica* extracts on cholangiocarcinoma cell proliferation and induction of apoptosis. J Herb Med. 35: 100582. 10.1016/j.hermed.2022.100582.

[cit0030] Chen SL, Yu H, Luo HM, Wu Q, Li CF, Steinmetz A. 2016. Conservation and sustainable use of medicinal plants: problems, progress, and prospects. Chin Med. 11: 37. 10.1186/s13020-016-0108-7. PMC4967523 27478496

[cit0031] Choudhari AS, Mandave PC, Deshpande M, Ranjekar P, Prakash O. 2020. Phytochemicals in cancer treatment: from preclinical studies to clinical practice. Front Pharmacol. 10: 1614. 10.3389/fphar.2019.01614. PMC7025531 32116665

[cit0032] Colak DK, Egeli U, Eryilmaz IE, Aybastier O, Malyer H, Cecener G, Tunca B. 2022. The anticancer effect of *Inula viscosa* methanol extract by miRNAs’ re-regulation: an *in vitro* study on human malignant melanoma cells. Nutr Cancer. 74: 211–224. 10.1080/01635581.2020.1869791 33570434

[cit0033] Csupor D, Widowitz U, Blazsó G, Laczkó-Zöld E, Tatsimo JS, Balogh Á, Boros K, Dankó B, Bauer R, Hohmann J. 2013. Anti-inflammatory activities of eleven *Centaurea* species occurring in the Carpathian Basin. Phytother Res. 27: 540–544. 10.1002/ptr.4754 22674731

[cit0034] Desai AG, Qazi GN, Ganju RK, El-Tamer M, Singh J, Saxena AK, Bedi YS, Taneja SC, Bhat HK. 2008. Medicinal plants and cancer chemoprevention. Curr Drug Metab 9(7): 581–591. 10.2174/138920008785821657 PMC4160808 18781909

[cit0035] De Souza LS, Puziol LC, Tosta CL, Bittencourt ML, Santa Ardisson J, Kitagawa RR, Filgueiras PR, Kuster RM. 2019. Analytical methods to access the chemical composition of an *Euphorbia tirucalli* anticancer latex from traditional Brazilian medicine. J Ethnopharmacol. 237: 255–265. 10.1016/j.jep.2019.03.041 30928500

[cit0036] De D, Panda SK, Ghosh U. 2023. Induction of apoptosis by ethanolic extract of leaf of *Dillenia pentagyna* Roxb. in A549 cells via NF-κB pathway. Phytomedicine Plus 3: 100368. 10.1016/j.phyplu.2022.100368.

[cit0037] Dehelean CA, Marcovici I, Soica C, Mioc M, Coricovac D, Iurciuc S, Cretu OM, Pinzaru I. 2021. Plant-derived anticancer compounds as new perspectives in drug discovery and alternative therapy. Molecules. 26: 1109. 10.3390/molecules26041109. PMC7922180 33669817

[cit0038] Deo SVS, Sharma J, Kumar S. 2022. GLOBOCAN 2020 report on global cancer burden: challenges and opportunities for surgical oncologists. Ann Surg Oncol. 29: 6497–6500. 10.1245/s10434-022-12151-6 35838905

[cit0039] El Yaagoubi OM, Lahmadi A, Bouyahya A, Filali H, Samaki H, El Antri S, Aboudkhil S. 2021. Antitumor effect of *Inula viscosa* extracts on DMBA-induced skin carcinoma are mediated by proteasome inhibition. Biomed Res Int. 2021: 6687589. 10.1155/2021/6687589. PMC8019636 33855081

[cit0040] Fatima N, Baqri SSR, Alsulimani A, Fagoonee S, Slama P, Kesari KK, Roychoudhury S, Haque S. 2021. Phytochemicals from Indian ethnomedicines: promising prospects for the management of oxidative stress and cancer. Antioxidants. 10: 1606. 10.3390/antiox10101606. PMC8533600 34679741

[cit0041] Ferlay J, Ervik M, Lam F, Colombet M, Mery L, Piñeros M, Znaor A, Soerjomataram I, Bray F. 2020. Global cancer observatory: cancer today. Lyon: International Agency for Research on Cancer.

[cit0042] Ferreira LG, Dos Santos RN, Oliva G, Andricopulo AD. 2015. Molecular docking and structure-based drug design strategies. Molecules. 20: 13384–13421. 10.3390/molecules200713384 PMC6332083 26205061

[cit0043] Gaidhani SN, Singh A, Kumari S, Lavekar GS, Juvekar AS, Sen S, Padhi MM. 2013. Evaluation of some plant extracts for standardization and anticancer activity. Indian J Tradit Knowl. 12: 682–687.

[cit0044] Gajarmal AA, Shantilal MS, Rath S. 2020. Ayurveda herbs in the flora of Punjab (India) with special reference to Bhavprakasha Nighantu – a review. J Phytopharmacol. 9: 67–75.

[cit0045] Gallo K, Kemmler E, Goede A, Becker F, Dunkel M, Preissner R, Banerjee P. 2023. SuperNatural 3.0 – a database of natural products and natural product-based derivatives. Nucleic Acids Res. 51: D654–D659. 10.1093/nar/gkac1008 PMC9825600 36399452

[cit0046] Gavamukulya Y, Abou-Elella F, Wamunyokoli F, El-Shemy HA. 2014. Phytochemical screening, anti-oxidant activity and *in vitro* anticancer potential of ethanolic and water leaves extracts of *Annona muricata* (Graviola). Asian Pac J Trop Med. 7: S355–S363. 10.1016/S1995-7645(14)60258-3. 25312150

[cit0047] Ghagane SC, Puranik SI, Kumbar VM, Nerli RB, Jalalpure SS, Hiremath MB, Neelagund S, Aladakatti R. 2017. *In vitro* antioxidant and anticancer activity of *Leea indica* leaf extracts on human prostate cancer cell lines. Integr Med Res. 6: 79–87. 10.1016/j.imr.2017.01.004 PMC5395687 28462147

[cit0048] Ginting B, Yahya M, Nurdin, Maulidna M, Murniana M, Safrina S. 2021. Evaluation of antioxidant and anticancer activity of *Myristica fragrans* Houtt. bark. Pharmacogn J. 13: 780–786.

[cit0049] Gospodinova Z, Bózsity N, Nikolova M, Krasteva M, Zupkó I. 2017a. Antiproliferative properties against human breast, cervical and ovarian cancer cell lines, and antioxidant capacity of leaf aqueous ethanolic extract from *Cotinus coggygria* Scop. Acta Med Bulg. 44: 20–25.

[cit0050] Gospodinova Z, Krasteva M, Manova V. 2017b. Effects of *Cotinus coggygria* extract on the transcriptional levels of histone deacetylase genes in breast cancer cells *in vitro*. Am J Pharm Health Res. 5: 60–69.

[cit0051] Gospodinova ZI, Zupkó I, Bózsity N, Manova VI, Georgieva MS, Todinova SJ, Taneva SG, Ocsovszki I, Krasteva ME. 2021. *Cotinus coggygria* Scop. induces cell cycle arrest, apoptosis, genotoxic effects, thermodynamic and epigenetic events in MCF7 breast cancer cells. Z Naturforsch. 76: 129–140. 10.1515/znc-2020-0087 32975208

[cit0052] Guchhait KC, Manna T, Barai M, Karmakar M, Nandi SK, Jana D, Dey A, Panda S, Raul P, Patra A, et al. 2022. Antibiofilm and anticancer activities of unripe and ripe *Azadirachta indica* (neem) seed extracts. BMC Complement Med Ther. 22: 1–18. 10.1186/s12906-022-03513-4 PMC8843028 35152903

[cit0053] Gupta N, Singh S, Chauhan D, Srivastava R, Singh VK. 2023. Exploring the anticancer potentials of polyphenols: a comprehensive review of patents in the last five years. Recent Pat Anticancer Drug Discov. 18: 3–10. 10.2174/1574892817666220512220036 35549878

[cit0054] Hossain MJ, Biswas S, Shahriar M, Chowdhury MM, Islam S, Ahsan CR, Auther C. 2018. Phytochemical screening, anti-microbial activity, antioxidant capacity and *in vivo* anticancer activity of *Lannea coromandelica* bark extracts. IOSR J Pharm Biol Sci. 13: 19–25.

[cit0055] Hussain N. 2020. Anticancer and antioxidant activities of *Cordia dichotoma* Forst. Int J Green Pharm. 14: 265–273.

[cit0056] Ibrahim MA, Cantrell CL, Jeliazkova EA, Astatkie T, Zheljazkov VD. 2020. Utilization of nutmeg (*Myristica fragrans Houtt*.) seed hydrodistillation time to produce essential oil fractions with varied compositions and pharmacological effects. Molecules 25: 565. 10.3390/molecules25030565. PMC7037852 32012955

[cit0057] Iliev I, Todorova K, Dimitrova M, Ivanov I. 2020. Effects of a *Cotinus coggygria* ethyl acetate extract on two human normal cell lines. Acta Morphol Anthropol. 27: 25–29.

[cit0058] Ingle SG, Gade AK, Hedawoo GB. 2024. Systematic review on phytochemicals structure and activity databases. Phytomedicine Plus 4: 100644. 10.1016/j.phyplu.2024.100644.

[cit0059] Jaiswal YS, Williams LL. 2017. A glimpse of Ayurveda – the forgotten history and principles of Indian traditional medicine. J Tradit Complement Med. 7: 50–53. 10.1016/j.jtcme.2016.02.002 PMC5198827 28053888

[cit0060] Jarial R, Thakur S, Sakinah M, Zularisam AW, Sharad A, Kanwar SS, Singh L. 2018. Potent anticancer, antioxidant and antibacterial activities of isolated flavonoids from *Asplenium nidus*. J King Saud Univ Sci. 30: 185–192.

[cit0061] Kalaivani S, Jayanthi S, Revathi K, Chandrasekaran R. 2024. Phytochemical profile of *Euphorbia hirta* plant extract and its *in vitro* anticancer activity against the liver cancer HepG2 cells. Vegetos. 37: 528–535.

[cit0062] Kashif M, Hwang Y, Kim WJ, Kim G. 2019. *In-vitro* morphological assessment of apoptosis induced by nimbolide; a limonoid from *Azadirachta indica* (neem tree). Iran J Pharm Res. 18: 846–859. 10.22037/ijpr.2019.2391 PMC6706709 31531067

[cit0063] Kaur A, Singh G, Sharma Y, Kumar M, Kumar A, Bala K. 2024. Assessing the potential of rosary pea (*Abrus precatorius L*.) derived aqueous seed extracts as anticancer agents and their phytoconstituents as COX-2 inhibitor: an *in-vitro* and *in-silico* approach. J Biomol Struct Dyn. 42: 9269–9282. 10.1080/07391102.2023.2251053 37649393

[cit0064] Khairunnisa K, Karthik D. 2014. Evaluation of *in-vitro* apoptosis induction, cytotoxic activity of *Hymenodictyon excelsum* (Roxb.) Wall. in Dalton’s lymphoma ascites (DLA) and lung fibroblast-mouse L929 cell lines. J Appl Pharm Sci. 4: 011–017. 10.7324/JAPS.2014.40803.

[cit0065] Kinghorn AD, De Blanco EJC, Lucas DM, Rakotondraibe HL, Orjala J, Soejarto DD, Oberlies NH, Pearce CJ, Wani MC, Stockwell BR, et al. 2016. Discovery of anticancer agents of diverse natural origin. Anticancer Res. 36: 5623–5637. 10.21873/anticanres.11146 PMC5098703 27793884

[cit0066] Kitdamrongtham W, Ishii K, Ebina K, Zhang J, Ukiya M, Koike K, Akazawa H, Manosroi A, Manosroi J, Akihisa T. 2014. Limonoids and flavonoids from the flowers of *Azadirachta indica* var. *siamensis*, and their melanogenesis-inhibitory and cytotoxic activities. Chem Biodivers. 11: 73–84. 10.1002/cbdv.201300266 24443427

[cit0067] Kopytko P, Piotrowska K, Janisiak J, Tarnowski M. 2021. Garcinol – a natural histone acetyltransferase inhibitor and new anti-cancer epigenetic drug. Int J Mol Sci. 22: 2828. 10.3390/ijms22062828. PMC8001519 33799504

[cit0068] Koul B, Koul B. 2019. Role of Ayurveda in cancer treatment. In: Herbs for Cancer Treatment. Springer, Singapore; p. 151–191.

[cit0069] Kour N, Qayum A, Lone BA, Singh SK, Sharma V, Gupta P. 2024. A novel carissic acid from fruits of *Carissa carandas* induced apoptotic cell death in A-549 cells following the activation of caspases. Nat Prod Res. 38: 1887–1897. 10.1080/14786419.2023.2232082 37449969

[cit0070] Krishnan HB, Kim S, Pereira AE, Jurkevich A, Hibbard BE. 2022. *Adenanthera pavonina*, a potential plant-based protein resource: seed protein composition and immunohistochemical localization of trypsin inhibitors. Food Chem X. 13: 100253. 10.1016/j.fochx.2022.100253. PMC9040029 35498991

[cit0071] Kumar S, Baldi A, Sharma DK. 2021. *In vitro* antioxidant assay guided *ex vivo* investigation of cytotoxic effect of phytosomes assimilating taxifolin rich fraction of *Cedrus deodara* bark extract on human breast cancer cell lines (MCF7). J Drug Deliv Sci Technol. 63: 102486. 10.1016/j.jddst.2021.102486.

[cit0072] Kumar S, Malhotra R, Kumar D. 2010. *Euphorbia hirta*: its chemistry, traditional and medicinal uses, and pharmacological activities. Pharmacogn Rev. 4: 58–61. 10.4103/0973-7847.65327 PMC3249903 22228942

[cit0073] Kumari S. 2022. Investigating the antioxidant and anticancer effect of alkaloids isolated from root extracts of *Berberis aristata*. Chem Data Collect. 37: 100805. 10.1016/j.cdc.2021.100805.

[cit0074] Lagoa R, Silva J, Rodrigues JR, Bishayee A. 2020. Advances in phytochemical delivery systems for improved anticancer activity. Biotechnol Adv. 38: 107382. 10.1016/j.biotechadv.2019.04.004. 30978386

[cit0075] Laskar YB, Mazumder PB. 2020. Insight into the molecular evidence supporting the remarkable chemotherapeutic potential of *Hibiscus sabdariffa* L. Biomed Pharmacother. 127: 110153. 10.1016/j.biopha.2020.110153. 32344257

[cit0076] Lautié E, Russo O, Ducrot P, Boutin JA. 2020. Unraveling plant natural chemical diversity for drug discovery purposes. Front Pharmacol. 11: 397. 10.3389/fphar.2020.00397. PMC7154113 32317969

[cit0077] Li CQ, Lei HM, Hu QY, Li GH, Zhao PJ. 2021. Recent advances in the synthetic biology of natural drugs. Front Bioeng Biotechnol. 9: 691152. 10.3389/fbioe.2021.691152. PMC8358299 34395399

[cit0078] Li F, Jiang T, Li Q, Ling X. 2017. Camptothecin (CPT) and its derivatives are known to target topoisomerase I (Top1) as their mechanism of action: did we miss something in CPT analogue molecular targets for treating human disease such as cancer? Am J Cancer Res. 7: 2350–2394. PMC5752681 29312794

[cit0079] Lim SH, Lee HS, Lee CH, Choi CI. 2021. Pharmacological activity of *Garcinia indica* (Kokum): an updated review. Pharmaceuticals 14: 1338. 10.3390/ph14121338. PMC8708457 34959738

[cit0080] Liu WC, Gong T, Zhu P. 2016. Advances in exploring alternative Taxol sources. RSC Adv. 6: 48800–48809.

[cit0081] Liu X, Zhao M, Wu K, Chai X, Yu H, Tao Z, Wang J. 2012. Immunomodulatory and anticancer activities of phenolics from emblica fruit (*Phyllanthus emblica L*.). Food Chem. 131: 685–690.

[cit0082] Liu Y, Jiang J, Zhang Z, Huang Q, Chen L, Huang Y, Zou X, Nie Y, Liu M, Zheng Y. 2024. Exploring the efficacy of *Phyllanthus emblica* L. based on association rule mining and multidimensional analysis. CyTA J Food. 22: 2293920. 10.1080/19476337.2023.2293920.

[cit0083] Mahata S, Pandey A, Shukla S, Tyagi A, Husain SA, Das BC, Bharti AC. 2013. Anticancer activity of *Phyllanthus emblica* Linn. (Indian gooseberry): inhibition of transcription factor AP-1 and HPV gene expression in cervical cancer cells. Nutr Cancer. 65: 88–97. 10.1080/01635581.2013.785008 23682787

[cit0084] Mangal M, Sagar P, Singh H, Raghava GP, Agarwal SM. 2013. NPACT: naturally occurring plant-based anti-cancer compound-activity-target database. Nucleic Acids Res. 41: D1124–D1129. 10.1093/nar/gks1047 PMC3531140 23203877

[cit0085] Manglani N, Vaishnava S, Dhamodaran P, Sawarkar H. 2014. *In vitro* and *in vivo* anticancer activity of leaf extract of *Barleria grandiflora*. Int J Pharm Pharm Res. 6: 70–72.

[cit0086] Mani ST, Rathinavel T, Ammashi S, Nasir Iqbal M. 2024. Polycyclic aromatic bioactive compounds from *Eclipta alba* and its anticancer potential against breast cancer target proteins: an antibreast cancer intervention through *in silico* and *in vitro* validations. Polycycl Aromat Compd. 44: 3313–3342.

[cit0087] Mazumder K, Biswas B, Raja IM, Fukase K. 2020. A review of cytotoxic plants of the Indian subcontinent and a broad-spectrum analysis of their bioactive compounds. Molecules. 25: 1904. 10.3390/molecules-25081904. PMC7221707 32326113

[cit0088] Meetei PA, Singh P, Nongdam P, Prabhu NP, Rathore RS, Vindal V. 2012. NeMedPlant: a database of therapeutic applications and chemical constituents of medicinal plants from north-east region of India. Bioinformation 8: 209–211. 10.6026/97320630008209 PMC3302005 22419844

[cit0089] Merlín-Lucas V, Ordoñez-Razo RM, Calzada F, Solís A, García-Hernández N, Barbosa E, Valdés M. 2021. Antitumor potential of *Annona muricata* Linn. an edible and medicinal plant in Mexico: *in vitro, in vivo*, and toxicological studies. Molecules 26: 7675. 10.3390/molecules26247675. PMC8707445 34946755

[cit0090] Mishra BA, Tomer V, Kumar A. 2024. Karonda (*Carissa carandas* L.): a miracle fruit with multi-faceted potential. J Agric Food Res. 18: 101417. 10.1016/j.jafr.2024.101417.

[cit0091] Moore BD, Andrew RL, Külheim C, Foley WJ. 2014. Explaining intraspecific diversity in plant secondary metabolites in an ecological context. New Phytol. 201: 733–750. 10.1111/nph.12526 24117919

[cit0092] Munro B, Vuong QV, Chalmers AC, Goldsmith CD, Bowyer MC, Scarlett CJ. 2015. Phytochemical, antioxidant and anticancer properties of *Euphorbia tirucalli* methanolic and aqueous extracts. Antioxidants. 4: 647–661. 10.3390/antiox4040647 PMC4712938 26783950

[cit0093] Mustafa K, Yu S, Zhang W, Mohamed H, Naz T, Xiao H, Liu Y, Nazir Y, Fazili ABA, Nosheen S, et al. 2021. Screening, characterization, and in vitro-ROS dependent cytotoxic potential of extract from *Ficus carica* against hepatocellular (HepG2) carcinoma cells. S Afr J Bot. 138: 217–226.

[cit0094] Naselli F, Tesoriere L, Caradonna F, Bellavia D, Attanzio A, Gentile C, Livrea MA. 2014. Anti-proliferative and proapoptotic activity of whole extract and isolated indicaxanthin from *Opuntia ficus-indica* associated with re-activation of the onco-suppressor p16INK4a gene in human colorectal carcinoma (Caco-2) cells. Biochem Biophys Res Commun. 450: 652–658. 10.1016/j.bbrc.2014.06.029 24937448

[cit0095] Neipihoi, Narzary B, Saikia S, Saikia S, Tamuli KJ, Sahoo RK, Dutta D, Bordoloi M. 2021. Anticancer and antimicrobial compounds from *Croton caudatus* Gieseler and *Eurya acuminata* DC: two edible plants used in the traditional medicine of the Kuki tribes. Nat Prod Res. 35: 6025–6029. 10.1080/14786419.2020.1815737 32936060

[cit0096] Nelson VK, Sahoo NK, Sahu M, Sudhan HH, Pullaiah CP, Muralikrishna KS. 2020. *In vitro* anticancer activity of *Eclipta alba* whole plant extract on colon cancer cell HCT-116. BMC Complement Med Ther. 20: 355. 10.1186/s12906-020-03118-9. PMC7681951 33225921

[cit0097] Neo SY, Siew YY, Yew HC, He Y, Poh KL, Tsai YC, Ng SL, Tan WX, Chong TI, Lim CSES, et al. 2023. Effects of *Leea indica* leaf extracts and its phytoconstituents on natural killer cell-mediated cytotoxicity in human ovarian cancer. BMC Complement Med Ther. 23: 79. 10.1186/s12906-023-03904-1. PMC10007844 36899361

[cit0098] Newman DJ, Cragg GM. 2020. Natural products as sources of new drugs over the nearly four decades from 01/1981 to 09/2019. J Nat Prod. 83: 770–803. 10.1021/acs.jnatprod.9b01285 32162523

[cit0099] Okoro EE, Maharjan R, Jabeen A, Ahmad MS, Azhar M, Shehla N, Zaman W, Shams S, Osoniyi OR, Onajobi FD, et al. 2021. Isoflavanquinones from *Abrus precatorius* roots with their antiproliferative and anti-inflammatory effects. Phytochemistry 187: 112743. 10.1016/j.phytochem.2021.112743. 33962136

[cit0100] Ozcan G, Dagdeviren Ozsoylemez O, Akman G, Khalilia W, Tezel BY, Karagoz A, Melikoglu G, Anil S, Kultur S, Sutlupinar N. 2016. Screening for antitumor activity of various plant extracts on HeLa and C4-I cell lines. J BUON. 21: 1552–1560. 28039722

[cit0101] Pai KSR, Srilatha P, Suryakant K, Setty MM, Nayak PG, Rao CM, Baliga MS. 2012. Anticancer activity of Berberis aristata in Ehrlich ascites carcinoma-bearing mice: a preliminary study. Pharm Biol. 50: 270–277. 10.3109/13880209.2011.599035 22085276

[cit0102] Pandey MM, Rastogi S, Rawat AKS. 2013. Indian traditional ayurvedic system of medicine and nutritional supplementation. Evid Based Complement Alternat Med. 2013: 376327. 10.1155/2013/376327. PMC3705899 23864888

[cit0103] Pathania S, Ramakrishnan SM, Bagler G. 2015. Phytochemica: a platform to explore phytochemicals of medicinal plants. Database 2015: bav075. 10.1093/database/bav075. PMC4529746 26255307

[cit0104] Potshangbam AM, Polavarapu R, Rathore RS, Naresh D, Prabhu NP, Potshangbam N, Kumar P, Vindal V. 2019. MedPServer: a database for identification of therapeutic targets and novel leads pertaining to natural products. Chem Biol Drug Des. 93: 438–446. 10.1111/cbdd.13430 30381914

[cit0105] Prasathkumar M, Anisha S, Dhrisya C, Becky R, Sadhasivam S. 2021. Therapeutic and pharmacological efficacy of selective Indian medicinal plants – a review. Phytomedicine Plus 1: 100029. 10.1016/j.phyplu.2021.100029.

[cit0106] Raaman N, Balasubramanian K. 2012. Antioxidant and anticancer activity of *Helicteres isora* dried fruit solvent extracts. J Acad Ind Res. 1: 148–152.

[cit0107] Radha MH, Laxmipriya NP. 2015. Evaluation of biological properties and clinical effectiveness of *Aloe vera*: a systematic review. J Tradit Complement Med. 5: 21–26. 10.1016/j.jtcme.2014.10.006 PMC4488101 26151005

[cit0108] Raghuvanshi D, Dhalaria R, Sharma A, Kumar D, Kumar H, Valis M, Kuča K, Verma R, Puri S. 2021. Ethnomedicinal plants traditionally used for the treatment of jaundice (icterus) in Himachal Pradesh in Western Himalaya – a review. Plants 10: 232. 10.3390/plants10020232. PMC7910824 33504029

[cit0109] Raghuvanshi D, Sharma K, Verma R, Kumar D, Kumar H, Khan A, Valko M, Alomar SY, Alwasel SH, Nepovimova E, Kuca K. 2022. Phytochemistry and pharmacological efficacy of *Cordia dichotoma* G. Forst. (Lashuda): a therapeutic medicinal plant of Himachal Pradesh. Biomed Pharmacother. 153: 113400. 10.1016/j.biopha.2022.113400. 36076525

[cit0110] Rahman MM. 2015. Evaluation of *Hymenodictyon excelsum* phytochemical’s therapeutic value against prostate cancer by molecular docking study. Jundishapur J Nat Pharm Prod. 10: e18216. 10.17795/jjnpp-18216. PMC4377060 25866716

[cit0111] Raina S, Sharma V, Sheikh ZN, Kour N, Singh SK, Zari A, Zari TA, Alharby HF, Hakeem KR. 2022. Anticancer activity of *Cordia dichotoma* against a panel of human cancer cell lines and their phytochemical profiling via HPLC and GCMS. Molecules. 27: 2185. 10.3390/molecules27072185. PMC9000789 35408583

[cit0112] Rasul HO, Aziz BK, Ghafour DD, Kivrak A. 2023. Screening the possible anti-cancer constituents of *Hibiscus rosasinensis* flower to address mammalian target of rapamycin: an in silico molecular docking, HYDE scoring, dynamic studies, and pharmacokinetic prediction. Mol Divers. 27: 2273–2296. 10.1007/s11030-022-10556-9 36318405

[cit0113] Rattanamaneerusmee A, Thirapanmethee K, Nakamura Y, Bongcheewin B, Chomnawang MT. 2018. Chemopreventive and biological activities of *Helicteres isora* L. fruit extracts. Res Pharm Sci. 13: 484–492. 10.4103/1735-5362.245960 PMC6288992 30607146

[cit0114] Ravishankar B, Shukla VJ. 2007. Indian systems of medicine: a brief profile. Afr J Tradit Complement Altern Med. 4: 319–337. 10.4314/ajtcam.v4i3.31226 PMC2816487 20161896

[cit0115] Reddy S, Subedi B, Guite N. 2023. Introduction: ethnomedicine and tribal healing practices in India: challenges and possibilities of recognition and integration. In: Ethnomedicine and Tribal Healing Practices in India: Challenges and Possibilities of Recognition and Integration. p. 1–31.

[cit0116] Regassa H, Sourirajan A, Kumar V, Pandey S, Kumar D, Dev K. 2022. A review of medicinal plants of the Himalayas with anti-proliferative activity for the treatment of various cancers. Cancers. 14: 3898. 10.3390/cancers14163898. PMC9406073 36010892

[cit0117] Rengasamy G, Venkataraman A, Veeraraghavan VP, Jainu M. 2018. Cytotoxic and apoptotic potential of *Myristica fragrans* Houtt. (mace) extract on human oral epidermal carcinoma KB cell lines. Braz J Pharm Sci. 54: e18028. 10.1590/s2175-97902018000318028.

[cit0118] Rosangkima G, Jagetia GC. 2015. *In vitro* anticancer screening of medicinal plants of Mizoram State, India, against Dalton’s lymphoma, MCF-7 and HeLa cells. Int J Recent Sci Res. 6: 5648–5653.

[cit0119] Rosangkima G, Rongpi T, Prasad SB. 2008. Role of glutathione and glutathione-related enzymes in the antitumor activity of *Dillenia pentagyna* in Dalton’s lymphoma-bearing mice. Int J Cancer Res. 4: 92–102.

[cit0120] Saeed W, Ismail T, Qamar M, Khan MZ, Ahmad N, Mubarak MS, Esatbeyoglu T. 2024. *Carissa carandas*: a multifaceted approach to health, wellness, and commerce. J Agric Food Res. 18: 101274. 10.1016/j.jafr.2024.101274.

[cit0121] Sak K. 2022. Anticancer action of plant products: changing stereotyped attitudes. Explor Target Antitumor Ther. 3: 423–427. 10.37349/etat.2022.00092 PMC9400775 36046223

[cit0122] Sathishkumar K, Chaturvedi M, Das P, Stephen S, Mathur P. 2022. Cancer incidence estimates for 2022 & projection for 2025: result from National Cancer Registry Programme, India. Indian J Med Res. 156: 598–607. 10.4103/ijmr.ijmr_1821_22 PMC10231735 36510887

[cit0123] Shaikh R, Pund M, Dawane A, Iliyas S. 2014. Evaluation of anticancer, antioxidant, and possible anti-inflammatory properties of selected medicinal plants used in Indian traditional medication. J Tradit Complement Med. 4: 253–257. 10.4103/2225-4110.128904 PMC4220503 25379467

[cit0124] Shalabi M, Khilo K, Zakaria MM, Elsebaei MG, Abdo W, Awadin W. 2015. Anticancer activity of *Aloe vera* and *Calligonum comosum* extracts separately on hepatocellular carcinoma cells. Asian Pac J Trop Biomed. 5: 375–381.

[cit0125] Shehata MG, Abu-Serie MM, Abd El-Aziz NM, El-Sohaimy SA. 2021. Nutritional, phytochemical, and *in vitro* anticancer potential of sugar apple (*Annona squamosa*) fruits. Sci Rep. 11: 6224. 10.1038/s41598-021-85772-8. PMC7973736 33737634

[cit0126] Shehna S, Sreelekshmi S, Remani PR, Padmaja G, Lakshmi S. 2022. Anti-cancer, anti-bacterial and anti-oxidant properties of an active fraction isolated from *Curcuma zedoaria* rhizomes. Phytomedicine Plus. 2: 100195. https://doi.org/10.1016/j.phyplu.2021.100195.

[cit0127] Shen Y, Sun Z, Shi P, Wang G, Wu Y, Li S, Zheng Y, Huang L, Lin L, Lin X, et al. 2018. Anticancer effect of petroleum ether extract from *Bidens pilosa L*. and its constituent’s analysis by GC-MS. J Ethnopharmacol. 217: 126–133. 10.1016/j.jep.2018.02.019 29454912

[cit0128] Shi X, Du R, Zhang J, Lei Y, Guo H. 2019. Evaluation of the anti-cancer potential of *Cedrus deodara* total lignans by inducing apoptosis of A549 cells. BMC Complement Altern Med. 19: 281. 10.1186/s12906-019-2682-6. PMC6813103 31651320

[cit0129] Shi Y, Zhang C, Li X. 2021. Traditional medicine in India. J Tradit Chin Med Sci. 8(Suppl 1): S51–S55. 10.1016/j.jtcms.2020.06.007.

[cit0130] Shimizu T, Torres MP, Chakraborty S, Souchek JJ, Rachagani S, Kaur S, Macha M, Ganti AK, Hauke RJ, Batra SK. 2013. Holy Basil leaf extract decreases tumorigenicity and metastasis of aggressive human pancreatic cancer cells in vitro and in vivo: potential role in therapy. Cancer Lett. 336: 270–280. 10.1016/j.canlet.2013.03.017 PMC3700662 23523869

[cit0131] Shine VJ, Latha PG, Suja SN, Anuja GI, Raj G, Rajasekharan SN. 2014. Ameliorative effect of alkaloid extract of *Cyclea peltata* (Poir.) Hook. f. & Thoms. roots (ACP) on APAP/CCl4 induced liver toxicity in Wistar rats and *in vitro* free radical scavenging property. Asian Pac J Trop Biomed. 4: 143–151. 10.1016/S2221-1691(14)60223-9 PMC3819483 25182286

[cit0132] Silva VAO, Rosa MN, Tansini A, Oliveira RJ, Martinho O, Lima JP, Pianowski LF, Reis RM. 2018. *In vitro* screening of cytotoxic activity of euphol from *Euphorbia tirucalli* on a large panel of human cancer-derived cell lines. Exp Ther Med. 16: 557–566. 10.3892/etm.2018.6244 PMC6090420 30112023

[cit0133] Singh G, Passsari AK, Singh P, Leo VV, Subbarayan S, Kumar B, Singh BP, Lalhlenmawia H, Kumar NS. 2017a. Pharmacological potential of *Bidens pilosa* L. and determination of bioactive compounds using UHPLC-QqQ LIT-MS/MS and GC/MS. BMC Complement Altern Med. 17: 492. 10.1186/s12906-017-2000-0. PMC5689161 29145848

[cit0134] Singh S, Kamble SN, Satdive RK, Fulzele DP. 2020. Heterologous overexpression of *Nothapodytes foetida* strictosidine synthase enhances levels of anti-cancer compound camptothecin in *Ophiorrhiza rugosa*. Plant Cell Tissue Organ Cult. 141: 67–76.

[cit0135] Singh VK, Arora D, Satija NK, Khare P, Roy SK, Sharma PK. 2017b. Intricatinol synergistically enhances the anticancerous activity of cisplatin in human A549 cells via p38 MAPK/p53 signalling. Apoptosis. 22: 1273–1286. 10.1007/s10495-017-1404-0 28756530

[cit0136] Singh VK, Thakur DC, Rajak N, Mishra A, Kumar A, Giri R, Garg N. 2023. The multi-protein targeting potential of bio active syringin in inflammatory diseases: using molecular modelling and in-silico analysis of regulatory elements. J Biomol Struct Dyn. 42: 1–12. 10.1080/07391102.2023.2273440. 37882327

[cit0137] Sophy AJR, Fleming AT, Vidhya R, Shankar KG, Rajesh BN. 2016. Cytotoxicity assessment of *Adenanthera pavonina* extracts in brine shrimp larvae and cancer cell lines. Int J Vet Sci. 5: 83–86.

[cit0138] Srivastava P, Srivastava A. 2015. *In vitro* anti-cancer activity of ethanolic extract of *Curcuma longa* (turmeric) in HEp-2 cell lines. Int J Eng Res Gen Sci. 3: 495–508.

[cit0139] Srivastava SK, Khan M, Khanuja SPS; Council of Scientific and Industrial Research CSIR. 2005. Process for isolation of anticancer agent camptothecin from *Nothapodytes foetida*. U.S. Patent 6,893,668.

[cit0140] Starok M, Preira P, Vayssade M, Haupt K, Salome L, Rossi C. 2015. EGFR inhibition by curcumin in cancer cells: a dual mode of action. Biomacromolecules. 16: 1634–1642. 10.1021/acs.biomac.5b00229 25893361

[cit0141] Su XZ, Miller LH. 2015. The discovery of artemisinin and the Nobel Prize in Physiology or Medicine. Sci China Life Sci. 58: 1175–1179. 10.1007/s11427-015-4948-7 PMC4966551 26481135

[cit0142] Sulaiman CT, Deepak M, Praveen TK, Lijini KR, Salman M, Sadheeshnakumari S, Balachandran I. 2023. Metabolite profiling and anti-cancer activity of two medicinally important *Euphorbia* species. Med Omics 7: 100018. 10.1016/j.meomic.2022.100018.

[cit0143] Sung H, Ferlay J, Siegel RL, Laversanne M, Soerjomataram I, Jemal A, Bray F. 2021. Global cancer statistics 2020: GLOBOCAN estimates of incidence and mortality worldwide for 36 cancers in 185 countries. CA Cancer J Clin. 71: 209–249. 10.3322/caac.21660 33538338

[cit0144] Takagi M, Tachi Y, Zhang J, Shinozaki T, Ishii K, Kikuchi T, Ukiya M, Banno N, Tokuda H, Akihisa T. 2014. Cytotoxic and melanogenesis-inhibitory activities of limonoids from the leaves of *Azadirachta indica* (Neem). Chem Biodivers. 11: 451–468. 10.1002/cbdv.201300348 24634075

[cit0145] Tomar R, Das SS, Balaga VKR, Tambe S, Sahoo J, Rath SK, Ruokolainen J, Kesari KK. 2024. Therapeutic implications of dietary polyphenols-loaded nanoemulsions in cancer therapy. ACS Appl Bio Mater. 7: 2036–2053. 10.1021/acsabm.3c01205 PMC11530091 38525971

[cit0146] Trivedi D, Jain P, Patel V, Kotasthane S, Das S. 2024. Herbal formulation: an Ayurvedic treatment for human diseases. In: Herbal Formulations, Phytochemistry and Pharmacognosy. Elsevier, p. 77–88.

[cit0147] Usmani S, Ahmad M, Hussain A, Arshad M, Ali M. 2018. Cellular oxidative stress and antiproliferative effects of *Cordia dichotoma* (Linn.) seeds extract and their fractions on human cervix epitheloid (HeLa) and human lung (A549) carcinoma cells. Eur J Integr Med. 21: 1–10.

[cit0148] Vallianou NG, Evangelopoulos A, Schizas N, Kazazis C. 2015. Potential anticancer properties and mechanisms of action of curcumin. Anticancer Res. 35: 645–651. 25667441

[cit0149] Vikas B, Anil S, Remani P. 2019. Cytotoxicity profiling of *Annona squamosa* in cancer cell lines. Asian Pac J Cancer Prev. 20: 2831–2840. 10.31557/APJCP.2019.20.9.2831 PMC6976856 31554384

[cit0150] Virdis P, Marchesi I, Fiorentino FP, Migheli R, Sanna L, Bordoni V, Pintore G, Galleri G, Muroni MR, Bagella L, et al. 2021. Tomentosin a sesquiterpene lactone induces antiproliferative and proapoptotic effects in human Burkitt lymphoma by deregulation of anti- and pro-apoptotic genes. Life (Basel) 11: 1128. 10.3390/life11111128. PMC8623649 34833004

[cit0151] Vivek-Ananth RP, Mohanraj K, Sahoo AK, Samal A. 2023. IMPPAT 2.0: an enhanced and expanded phytochemical atlas of Indian medicinal plants. ACS Omega. 8: 8827–8845. 10.1021/acsomega.3c00156 PMC9996785 36910986

[cit0152] Wang G, Wang J, Du L, Li F. 2015. Effect and mechanism of total flavonoids extracted from *Cotinus coggygria* against glioblastoma cancer *in vitro* and *in vivo*. BioMed Res Int. 2015: 856349. 10.1155/2015/856349. PMC4628721 26557705

[cit0153] Wang J, Rani N, Jakhar S, Redhu R, Kumar S, Kumar S, Kumar S, Devi B, Simal-Gandara J, Shen B, Singla RK. 2023. *Opuntia ficus-indica* (L.) Mill. – anticancer properties and phytochemicals: current trends and future perspectives. Front Plant Sci. 14: 1236123. 10.3389/fpls.2023.1236123. PMC10582960 37860248

[cit0154] Wang X, Zhang H, Chen X. 2019. Drug resistance and combating drug resistance in cancer. Cancer Drug Resist. 2: 141–160. 10.20517/cdr.2019.10 PMC8315569 34322663

[cit0155] Weerapreeyakul N, Junhom C, Barusrux S, Thitimetharoch T. 2016. Induction of apoptosis in human hepatocellular carcinoma cells by extracts of *Lannea coromandelica* (Houtt.) Merr. and *Diospyros castanea* (Craib) Fletcher. Chin Med. 11: 19. 10.1186/s13020-016-0091-z. PMC4841969 27110278

[cit0156] Widodo N, Puspitarini S, Widyananda MH, Alamsyah A, Wicaksono ST, Masruri M, Jatmiko YD. 2022. Anticancer activity of *Caesalpinia sappan* by downregulating mitochondrial genes in A549 lung cancer cell line. Research 11: 169. 10.12688/f1000research.76187.2. PMC9468624 36128561

[cit0157] Yan D, Zheng G, Wang C, Chen Z, Mao T, Gao J, Yan Y, Chen X, Ji X, Yu J, Mo S. 2022. HIT 2.0: an enhanced platform for Herbal Ingredients’ Targets. Nucleic Acids Res. 50: D1238–D1243. 10.1093/nar/gkab1011 PMC8728248 34986599

[cit0158] Yang D, Zhang X, Zhang W, Rengarajan T. 2018. Vicenin-2 inhibits Wnt/β-catenin signaling and induces apoptosis in HT-29 human colon cancer cell line. Drug Des Devel Ther. 12: 1303–1310. 10.2147/DDDT.S149307 PMC5965372 29849451

[cit0159] Yang L, Xie J, Almoallim HS, Alharbi SA, Chen Y. 2021. Tomentosin inhibits cell proliferation and induces apoptosis in MOLT-4 leukemia cancer cells through the inhibition of mTOR/PI3K/Akt signaling pathway. J Biochem Mol Toxicol. 35: 22719. 10.1002/jbt.22719. 33501684

[cit0160] Yen GC, Chen CS, Chang WT, Wu MF, Cheng FT, Shiau DK, Hsu CL. 2018. Antioxidant activity and anticancer effect of ethanolic and aqueous extracts of the roots of *Ficus beecheyana* and their phenolic components. J Food Drug Anal. 26: 182–192. 10.1016/j.jfda.2017.02.002 PMC9332673 29389554

[cit0161] Yi F, Li L, Xu LJ, Meng H, Dong YM, Liu HB, Xiao PG. 2018. *In silico* approach in reveal traditional medicine plants pharmacological material basis. Chin Med. 13: 33. 10.1186/s13020-018-0190-0. PMC6006786 29946351

[cit0162] Zahara K, Bibi Y, Masood S, Nisa S, Sher A, Ali N, Kumar S, Qayyum A, Ahmed W, Sami R, Al-Mushhin AA. 2022. Isolation and identification of bioactive compounds from *Bidens* spp. using HPLC-DAD and GC-MS analysis and their biological activity as anticancer molecules. Molecules. 27: 1927. 10.3390/molecules27061927. PMC8950169 35335292

[cit0163] Zhang RZ, Yu SJ, Bai H, Ning K. 2017. TCM-Mesh: the database and analytical system for network pharmacology analysis for TCM preparations. Sci Rep. 7: 2821. 10.1038/s41598-017-03039-7. PMC5460194 28588237

[cit0164] Zhang Y, Wan Y, Huo B, Li B, Jin Y, Hu X. 2018. Extracts and components of *Ficus carica* leaves suppress survival, cell cycle, and migration of triple-negative breast cancer MDA-MB-231 cells. OncoTargets Ther. 11: 4377–4386. 10.2147/OTT.S171601 PMC6067789 30100743

