# Supplementary material for: Cytotoxic plants from the Indian subcontinent: promising reservoir of anticancer agents
Source: BioTechnologia (Pozn). 2026 Aug 20;107(3):283–302. doi: 10.5114/bta/221149 (PMC13725477; doi:10.5114/bta/221149)
Supplement: Supplementary file 1 [file BTA-107-3-221149-s1.pdf]

Supplementary Table 1. List of plants with their phytochemicals, target cancer cell lines and mechanism of action

Plant name	Active compounds	Cancer type	Target cell lines	Mechanism	Study type	Evidence level	References
Abrus precatorius	Tannic acid, Rutin, Abruquinone M & N	Cervical, colon, oral, lung	HeLa, Hep2C, Caco-2, CAL-27, NCI–H460	COX-2 inhibition, TNF-α inhibition, Oxidative burst induction	*In vitro, in silico*	Preliminary	(Kaur *et al.* 2022; Kaur *et al.* 2024; Okoro *et al.* 2021)
Adenanthera pavonina	Not specified	Breast, lung, kidney, prostate	MCF-7, NCI-H460, 786–0, PC-3	Antiproliferati ve effect (enzymatic extract)	*In vitro*	Preliminary	(Chauhan *et al.* 2015; Sophy *et al.* 2016)
Aloe vera	Not specified	Liver	Hep-G2	Antiprolifera-tive, blocking signal transduction	*In vitro*	Preliminary	(Shalabi *et al.* 2015; Radha and Laxmipriya 2015)
Annona muricata	Acetogenins	Breast, prostate	MDA, SK-BR-3, EACC, BPH-1, 4T1	ATP synthase inhibition, Apoptosis induction	*In vitro, in vivo* (mouse)	Intermediate	(Gavamukulya *et al.* 2014; Asare *et al.* 2015; Merlin-Lucas *et al.* 2021)
Annona squamosa	Apigenin, rhamnetin, and eupafolin	Oral, lung, chronic myeloid leukemia, kidney, breast	MCF-7, HepG-2, Caco-2, and PC3	Cytotoxic effect on cancer cells	*In vitro*	Preliminary	(Shehata *et al.* 2021; Vikas *et al.* 2019)
Arbutus andrachne	Not specified	Breast, colon, lung, pancreas, prostate, skin	T47D, MCF-7, HRT-18, Caco-2, A375.S2, and WM 361A	Cytotoxic effect	*In vitro*	Priliminary	(Abu-rish *et al.* 2016)
Aristolochia ringens	Not specified	Leukemia, lung, prostate, cervical	A431, A549, PC-3, HeLa, and THP-1	Inhibition of tumor growth	*In vitro*	Priliminary	(Akindele *et al.* 2015; Daoudi *et al.* 2013)
Asplenium nidus	Quercetin-7-O-rutinoside and gliricidin-7-O-hexoside	Cervical, liver	HeLa and Hep-G2 cells	Cytotoxic effect	*In vitro*	Preliminary	(Jarial *et al.* 2018)
Azadirachta indica	Nimbin, nimbidol, nimbidin, and nimbolide	Breast , lung, stomach, leukemia	SK-BR-3, A-549, AZ-521 and HL-60	Apoptosis, by high expression of caspase 3, 8, and 9	*In vitro*	Preliminary	(Takagi *et al.* 2014; Kitdamrongtham *et al.* 2014; Kashif *et al.* 2019)
Barleria grandiflora	Not specified	Lung, lymphoma	DLA and Vero cell lines	Cytotoxic effect	*In vitro, in vivo* (mouse)	Intermediate	(Manglani *et al.* 2014)
Berberis aristata	Thalifendine, jatrorrhizine, and berberrubine	Ovary, lungs, oral	A 2780, Hop62 and DWD	Cytotoxic effect	*In silico, in vitro, in vivo* (mouse)	Intermediate	(Gaidhani *et al.* 2013; Pai *et al.* 2012; Kumari, 2022)

Biancaea sappan	Caesalpinia, and brazalin	Lung, breast	MCF-7 and A549	Exhibit DNA strand scission activity, inhibition of anti-apoptotic gene Bcl-2	*In silico, in vitro*	Preliminary	(Bukke *et al.* 2018; Widodo *et al.* 2022)
Bidens pilosa	Phenyl-1,3,5-heptatriyne, 2,4-di-tert-butylphenyl	Lung, cervical, liver	A-549, HeLa, and HepG-2	Cytotoxic effect	*In vitro, in vivo* (brine shrimp)	Intermediate	(Shen *et al.* 2018; Zahara *et al.* 2022)
Calligonum cosmosus	Kaempferol, catechin	Liver	Hep-G2	Apoptotic activity by declining Bcl-2 protein	*In vitro*	Preliminary	(Shalabi *et al.* 2015; Alehaideb *et al.* 2020)
Carissa carandas	Uvaol, 23-hydroxy ursolic acid, and 27-O-Z-p-coumaroyl ursolic acid	Liver, lung, bone, cervical, ovary, breast	NCI-H460, HT-29, MCF-7, and A-549	Chromatin condensation and decreased mitochondrial membrane potential by activating apoptosis	*In vitro*	Preliminary	(Bano *et al.* 2022; Kour *et al.* 2024)
Cedrus deodara	Not specified	Breast, ovary, pancreas	A-549, MCF-7, PC-3, A 2780, Mia-Pa-Ca-2	Cytotoxic effect	*In vitro*	Preliminary	(Shi *et al.* 2019; Kumar *et al.* 2021; Gaidhani *et al.* 2013)
Cenchrus ciliaris	Not specified	Liver, breast, cervical, lung, colon	HepG-2, A-549, MCF-7, HeLa, HCT-116 and Caco-2	Cytotoxic effect	*In vitro*	Preliminary	(Alothman *et al.* 2018)
Centaurea antiochia	Not specified	Cervical	HeLa	Cytotoxic effect	*In vitro*	Preliminary	(Csupor *et al.* 2013; Ozcan *et al.* 2016)
Cordia dichotoma	Caffeic acid, cis- vaccenic acid, ferulic acid, gallic acid, palmitic acid, and vanillic acid	Breast, lung, colon, and pancreas	SH-SY5Y, N2A, U-251, MCF-7, MDA-MB-231, A-549, SW-620, HCT-116, MIA PaCa-2, and PANC-1	Cytotoxic effect	*In vitro, in vivo* (Swiss albino mice)	Intermediate	(Hussain, 2020; Usmani *et al.* 2018)
Cotinus coggygria	Not specified	Breast, cervical, ovary	MCF-7, T47D, HeLa, A2780	Inhibit fibroblast activation protein α, Akt along with ERK expression (PI3K/Akt-ERK pathway)	*In vitro*	Preliminary	(Raina *et al.* 2022; Usmani *et al.* 2018)
Croton caudatus	β-sitosterol, *(E)*-dodec-3-en-1-ol and tricosan-1-ol, octacosanoic acid	Cervical, breast, lung	A-549, MCF-7, HeLa	Cytotoxic effect	*In vitro*	Preliminary	(Nepihoi *et al.* 2021; Rosangkima and Jagetia 2015)

Curcuma longa	Curcumin	Breast, colon, hepatic, prostate, leukemia	HepG-2, MCF-7, others	Multiple pathways (NF-κB, p53, apoptosis)	Clinical, *in vitro*	Advanced	(Shehna *et al.* 2022; Srivastva and Srivastava, 2015; Vallianou *et al.* 2015; Starok *et al.* 2015)
Cyclea peltata	Fangchinoline, tetrandrine	Breast, pancreas	MDA-MB-231 and PANC-1	Apoptosis through ROS generation	*In vitro*	Preliminary	(Shine *et al.* 2014; Chandrashekar *et al.* 2019)
Delphinium staphisagria	Not specified	Cervical, breast, lung, colon	A-549, MCF-7, HT-29, and HeLa	Apoptosis	*In vitro*	Preliminary	(Akgun *et al.* 2022)
Dillenia pentagyna	Not specified	Bone, lung, breast, cervical	U2OS, A-549, MCF-7 and HeLa	Cytotoxicity, apoptosis and depletion of glutathione	*In vitro, in vivo*	Intermediate	(De *et al.* 2023; Rosangkima *et al.* 2008)
Eclipta alba	Coumestan, wedelolactone, and β-sitosterol	Breast, renal, colon, prostate	MCF-7, HCT-116, PC-3, RCC-45	Mitochondrial apoptotic pathways	*In silico, in vitro, in vivo*	Intermediate	(Nelson *et al.* 2020; Arya *et al.* 2015; Mani *et al.* 2024)
Euphorbia hirta	β-amyrin, β-sitosterol, euphorbin A, B, C, and D; gallic acid, heptacosane, kaempferol, n-nonacosane, protocatechuic acid, quercetin, rutin, 1,3,4,6-tetra-O-galloyl-β-D-glucose, 2,4,6-tri-O-galloyl-β-D-glucose, and 24-methylene cycloartenol	Liver	HepG-2	Inhibit cell proliferation and apoptosis	*In vitro, in vivo* (mice)	Intermediate	(Kumar *et al.* 2010; Kalaivani *et al.* 2024)
Euphorbia tirucalli	Euphol and tirucallol	Pancreas	MiaPaCa-2	Cytotoxic effect	*In vitro*	Preliminary	(Munro *et al.* 2015; De Souza *et al.* 2019; Silva *et al.* 2018)
Ficus beecheyana	Caffeic acid, gallic acid, and rutin	Leukemia	HL-60	Increased expression of Bad, Bak, and Bax genes	*In vitro*	Preliminary	(Yen *et al.* 2018)
Ficus carica	β-bourbonene	Breast, colon, liver	MDA-MB-231, Hep-2, HepG-2, MCF-7, and Caco-2	Reducing AMPKa, CREB, ERK, and GSK-3α/β expression	*In vitro*	Preliminary	(Al Ghalban *et al.* 2021; Gurung *et al.* 2021; Mustafa *et al.* 2021; Zhang *et al.* 2018)

Garcinia indica	Cyanidin-3-glucoside, cyanidin-3-sambubioside, garcinol, and hydroxy-citric acid	Esophageal, gastric, colorectal, pancreatic, liver	DU145, MDA-MB-231, and BxPC-3	Inhibits histone acetyltransferases and disrupts the regulation of microRNAs	*In vitro, in vivo* (Xenograft mice model)	Intermediate	(Kopytko *et al.* 2021; Ahmad *et al.* 2012)
Helicteres isora	Isocucurbitacin B and cucurbitacin B	Lung, colon	NCI-H460, RCM-1	Cytotoxic effect	*In vitro*	Preliminary	(Raaman and Balasbramanian, 2012; Rattanamaneeru-smee *et al.* 2018)
Hibiscus rosa-sinensis	Stigmastadienol, lupeol, and taraxasterol acetate	Macrophage cancer cells, testicular cell line	RAW264.7, Tera-1	Cytotoxic effect	*In vitro, in silico*	Preliminary	(Al-Saily *et al.* 2018; Rasul *et al.* 2023)
Hibiscus sabdariffa	Delphinidin-3-sambubioside and protocatechuic acid	Leukemia	HL-60	Increasing SOD activity and decreasing MDA levels	*In vitro, In vivo* (Wister rat)	Intermediate	(Bassong *et al.* 2022)
Hymenodictyon excelsum	Anthraquinone and coumarin phytochemicals	Mouse cancer cell line, prostate	L-929 and DLA cells	Fragmentation of DNA	*In vitro*	Preliminary	(Chakraborty *et al.* 2017; Rahman, 2015)
Hypericum kotschyanum	Not specified	Cervical	HeLa	Cytotoxic effect	*In vitro*	Preliminary	(Artun *et al.* 2016)
Inula viscosa	Tomentosin	Human Burkitt's lymphoma, skin	MM cells, A2058, and MeWo	Apoptosis (by downregulation of CDKN1A and BCL2A1 and upregulation of PMAIP1 gene), reduction of miRNA, cell cycle arrest, inhibition of JAK/STAT and PI3K/AKT genes	*In vitro, in vivo* (mice)	Intermediate	(Colak *et al.* 2022; El Yaagoubi *et al.* 2021; Virdis *et al.* 2021; Yang *et al.* 2021)
Lagenaria siceraria	Cucurbitacin I	Colon, breast	HT-29 and MCF-7	Apoptosis	*In vitro*	Preliminary	(Attar and Ghane, 2019; Attar and Ghane, 2018)
Lannea coromandelica	2-Palmitoylglycerol, hexadecanoic acid and octadecenoic acid	Gastric cancer cell, liver	HepG-2	Cytotoxic effect	*In vitro, in vivo* (mice)	Intermediate	(Hossain *et al.* 2018; Weerapreeyakul *et al.* 2016)
Leea indica	Not specified	Prostate, ovarian	DU-145, PC-3, SK-OV-3 and OVCAR-5	Lowering production of TNF-α and IL-1β	*In vitro*	Preliminary	(Ghagane *et al.* 2017; Neo *et al.* 2023)
Moullava digyna	Intricatinol	Lung	A-549	Activates the p38 MAPK/p53	*In vitro*	Preliminary	(Singh *et al.* 2017)
Myristica fragrans	Myristicin, 6′-methyl-(7hydroxy-8-methylbut-9-en)-3,2′-dimethoxybiphenyl-4,5-diol)	Breast	MCF-7	Cytotoxic effect	*In vitro*	Preliminary	(Rengasamy *et al.* 2018; Ginting 2021)

Ocimum sanctum	Vicenin-2	Breast, colon	MCF-7 and HT-29	Increased expression of *Bid*, *Bax/Bcl-2* ratio, and *p53* genes, downregulation of genes like *p65*, *FAK*, and activated *ERK-1/2*	*In vitro*	Preliminary	(Manaharan *et al.* 2016; Shimizu *et al.* 2013; Yang *et al.* 2018)
Opuntia ficus-indica	Betanin, catechin, gallic acid, naringenin, naringin, protocatechuic acid, quercetin, and quinic acid	Chronic myeloid leukemia	K562	Apoptosis (demethylation of promoter of p16INK4a)	*In vitro*	Preliminary	(Naselli *et al.* 2014)
Phyllanthus emblica	Geraniin, glucopyranoside, isocorilagin, kaempferol, kaempferol 3-β-d-glucopyranoside, quercetin and rutin	Human embryonic lung fibroblast, breast, cervical	HELF, MCF-7 and HPV oncogenes	Apoptosis (increasing *caspase-3*, *-9*, *Bax*, and *p53* and reducing *Bcl-2* transcription)	*In vitro, in vivo*	Intermediate	(Liu *et al.* 2012; Liu *et al.* 2024; Chekdaengphano *et al.* 2022)

Supplementary Table 2.

Plant name	Active compounds	Cancer type	Target cell lines	Mechanism	Study type	Evidence level	References
Piper betle	Hydroxychavicol	Bone, Oral	MG63, Ca9-22 and Cal-27	Apoptosis (downregulating Bcl-xl, Bcl-2; ErbB2 and ErbB3 expression, and upregulating Bad, Bax gene; p38 MAPK)	*In vitro, in silico*	Preliminary	(Vinusri *et al.* 2022; Lee *et al.* 2022)
Portulaca oleracea	Oleraciamide D	Neuroblastoma, glioblastoma	SH-SY5Y, U-87	Inhibits NF-κB	*In vitro*	Preliminary	(Zhao *et al.* 2018; Rahimi *et al.* 2019
Ricinus communis	p-Epigallocatechin, coumaric acid, ricinine, and ricinoleic acid	Breast, cervical, liver, pancreatic, melanoma	MCF-7, HeLa HepG-2, PANC-1 MeWo	Promoting pro-apoptotic protein activity and inducing DNA damage	*In vitro*	Preliminary	(Majumder *et al.* 2019)
Saraca asoca	Phytoestrogens – β-sitosterol, quercetin, and kaempferol	Colon, breast, skin	HT-29, MCF-7 & MDA-MB-231, B16-F10	Cytotoxic effect	*In vitro, in silico*	Preliminary	(Roy *et al.* 2021; Pareeth *et al.* 2024)
Saurauia roxburghii Wall.	Not specified	Lung, breast	A431 and C6 cells	Cytotoxic effect	*In vitro*	Preliminary	(Ahmed *et al.* 2016; Fabelico, 2020)
Tabernaemontana divaricata	Tabernaemontanine and coronaridine	Colon, breast	502713, MCF-7, and MOLT4	Inhibiting the unwinding of supercoiled DNA	*In vitro, in vivo* (mice)	Intermediate	(Parthiban *et al.* 2015; Bhagat and Kumar 2021; Doshi *et al.* 2017; Santhi 2020)
Teucrium polium	Dihydrosamidin, cepharantine, 11Z octadecadienoic acid, 13R-hydroxy-9E, and valtratum	Lung, liver, prostate, breast	A-549, H322 and MDA-MB-231	Cytotoxic effect	*In vitro, in silico, in vivo* (mice)	Intermediate	(Nikodijević *et al.* 2016; Movahedi *et al.* 2014; Noumi *et al.* 2020; Alzeer *et al.* 2015)
Thymus vulgaris	Carvacrol	Osteosarcoma, prostate	1433B, PC-3	Cytotoxic effect (*via* upregulating ROS), suppressing Notch signalling	*In vitro*	Preliminary	(Khan *et al.* 2019; Zhang *et al.* 2022)
Tillandsia recurvata	Not specified	Breast, brain, neuroblastoma, leukemia, skin	MDA-MB-231, U87 MG, IMR-32, MV4-11, A375	Apoptosis (activation of caspase 3/7)	*In vitro*	Preliminary	(Lowe *et al.* 2012; Lowe *et al.* 2017)
Tribulus terrestris	Naurigenin	Breast, lung	MCF-7 and A549	Apoptosis (activation of caspase-3, suppression of Bcl-2)	*In vitro, in silico*	Preliminary	(Allshabi *et al.* 2022; Patel *et al.* 2019; Patel *et al.* 2021)

Vitis vinifera	Not specified	Colon, skin	HT-29, A375 and SK-MEL	Apoptosis	*In vitro*	Preliminary	(Sasikumar *et al.* 2020; Selma *et al.* 2021; Aghbali *et al.* 2013)
Withania coagulans	Withaferin A	Cerical, skeletal muscle, breast, rat tumor cells	HeLa, RD, MCF-7, MDA-MB-231, INS-1 and RG2	Apoptosis, cell cycle arrest at G2/M phase	*In vitro, In vivo* (mice)	Intermediate	(Maqsood *et al.* 2018; Ahmad *et al.* 2017; Gopinath *et al.*2017)
Withania somnifera	Withaferin A, 16β-acetoxy-6α,7α-epoxy-5α-hydroxy-1-oxowitha-2,17(20), 24-trienolide	CNS, breast, lung, colon, prostate	SF-268, MCF-7, NCI-H460, HCT-116, WRL-68, Caco-2, and PC-3	Cytotoxic effect	*In vitro, in vivo* (mice)	Intermediate	(Tewari *et al.* 2022; Saggam *et al.* 2020; Prasad *et al.* 2021; Siddique *et al.* 2014)
Zea mays	Maysin	Prostate	PC-3,	Apoptosis, DNA damage, downregulation of pro-caspase-3 and Bcl-2	*In vitro, in vivo* (mice)	Intermediate	(Lee *et al.* 2014; Guo *et al.* 2017; Hwang *et al.* 2018; Al-Oqail *et al.* 2019)
Zingiber officinale	Not specified	Breast, cervical	MDA-MB-231, HeLa	Apoptosis (upregulation of Bax and downregulation of Bcl-X, Bcl-2, cyclin D1, NF-κB, and CDK-4)	*In vitro*	Preliminary	(Park *et al.* 2014; Ansari *et al.* 2016)

***Piper betle*:**

Piper betle L. is classified under the *Piperaceae* family, native to Southeast Asia. The leaves of *P. betle* possess numerous therapeutic properties like antioxidant, antimicrobial, anti-inflammatory, and anticancer effects, with many well-known compounds including eugenol, hydroxychavicol, chlorophyll, and β-carotene (Looi *et al.* 2020). Hydroxychavicol has been determined as the main phytochemical exerting anticancer properties among these components. A recent study found that hydroxychavicol, a key compound derived from the leaves of *P. betle* L., demonstrated promising cytotoxic efficiency towards the MG63 bone cancer cells. Furthermore, it confirmed that hydroxychavicol interacts with 16 tested cancer targets via molecular docking (Vinusri *et al.* 2022). Another study found that the stem extract of this plant inhibits cell growth and triggers apoptosis in oral cancer cell lines Ca9-22 and Cal-27 by downregulating Bcl-xl and Bcl-2 while enhancing Bad and Bax levels, alongside activating p38 MAPK and suppressing ErbB2 and ErbB3 expressions (Lee *et al.* 2022).

***Portulaca oleracea* L.:**

Portulaca oleracea L., is classified under the family *Portulacaceae* and also known as Loni or Lonna in Ayurveda. This plant has been widely applied for treating a variety of health issues, including headaches, diabetes, ulcers, diarrhea, urinary infections, kidney and cardiovascular diseases. *P. oleracea* L. is well known to contain several important phytochemicals, including alkaloids, anthocyanins, betalains, catecholamines, flavonoids, lignans, phenolic acids, and terpenoids. It is also a rich source of ascorbic acid, β-carotene, glutathione, tocopherols, omega-3 and omega-6 fatty acids (Kumar *et al.* 2022). A recent study reported that oleraciamide D from *P. oleracea* L. exhibits anticancer efficacy towards neuroblastoma SH-SY5Y cells (Zhao *et al.* 2018). Research by Rahimi *et al.* in 2019 found that *P. oleracea* L. leaf extract inhibits NF-κB in a concentration- and time-dependent manner, showcasing anticancer effects on the glioblastoma U-87 cell line (Rahimi *et al.* 2019).

***Ricinus communis* L.:**

Ricinus communis L. belongs to the family *Euphorbiaceae*, generally referred to as the castor plant. It is indigenous to India and can be found across the entire country. Research has shown that seeds, fruit, and leaves of *R. communis* are used in traditional medicine to treat various ailments, including microbial infections, warts, gastritis, constipation, and paralysis. In addition, extensive cancer research has shown its promising anticancer effects against breast, cervical, liver, pancreatic, and melanoma cancers (Abomughaid *et al.* 2024). Recently, the fruit extract from *R. communis* was identified with four key bioactive compounds, namely p-epigallocatechin, coumaric acid, ricinine, and ricinoleic acid, which significantly contribute to anticancer effectiveness and restrict breast cancer cell migration, particularly in triple-negative MDA-MB-231 and estrogen-positive MCF-7 cells. It significantly reduced metastasis by decreasing matrix metalloproteinases (MMPs)-2 and -9 levels while promoting pro-apoptotic protein activity and inducing DNA damage. The study showed a notable decrease in tumor progression in a syngeneic mouse model (Majumder *et al.* 2019).

Saraca asoca* (Roxb.) Willd*:

Saraca asoca (Ashoka) is sacred evergreen tree in India known for its medicinal properties, widely distributed from Indo-China to West Malesia. Research by Jinu *et al.* (2015) highlights its antioxidant, antitumor, anti-oxytocic, anti-menorrhagic, and antimicrobial activities (Jinu and Jayabaskaran 2015). *S. asoca* bark extracts demonstrated notable cytotoxic effects towards the HT-29 cell line, evaluated through MTT and SRB assays (Jeenathunisa and Rajan 2021). *S. asoca* bark extract demonstrated notable antioxidant and cytotoxic effects towards breast cancer cell lines MCF-7 and MDA-MB-231, with no toxic effects on normal human cells (HEK-293) (Yadav *et al*. 2015). Another study evaluated the antiproliferative effects of this plant's flower extract at concentrations of 10 to 60 µg/ml against B16-F10 skin cancer cell line. At 60 µg/ml, it exhibits proapoptotic and anticancer effiicts (Roy *et al.* 2021). Recent findings identified three phytoestrogens, including β-sitosterol, quercetin, and kaempferol, that exhibit significant cytotoxicity towards those breast cancer cell lines that express estrogen receptor beta (Erβ), specifically MDAMB-231 and SKBR3, while showing no cytotoxic effects onMCF-7 with estrogen receptor alpha (ERα), even at higher concentrations. Furthermore, the docking results demonstrated that phytoestrogens, particularly quercetin, bind more effectively to ERβ than to Erα (Pareeth *et al.* 2024).

***Saurauia roxburghii* Wall.:**

Saurauia roxburghii is classified under the family *Actinidiaceae*, and found in the eastern and southeastern parts of Asia. It has been used as ethnomedicine by the native population of these regions to treat numerous ailments such as boils, eczema, stomach/digestion-related issues, piles, etc. (Nahrin *et al.* 2020). The compounds extracted from this plant have pharmacological

effects, including antimicrobial, anticancer, antioxidants, antiviral etc., and fit into two main classes, steroids and terpenes (Ahmed *et al.* 2016). The fruit of the plant possesses a higher phenolic content than that of leaves. Still, leaf extracts displayed cytotoxicity against brine shrimps, thus, promoting the idea of the pharmacological efficacy of this plant as a source of anticancer biomolecules (Fabelico 2020). The crude and pure compounds from the leaf extract displayed possible cytotoxic activities, supporting its use in ethnomedicine (Ahmed *et al.* 2016). Since not much work has been done on this plant's anticancer potential, the above data advocate that it is a high source of such compounds and may provide a roadmap for exploring its pharmacologically important components in the future.

Tabernaemontana divaricata (L.) R.Br. ex Roem. & Schult.:

Tabernaemontana divaricata, also known as *Ervatamia divaricata*, is a significant medicinal plant from the family *Apocynaceae*. It boasts a range of medicinal properties, including antioxidant, antimicrobial, anti-parasitic, anti-inflammatory, and anticancer. Recent, studies have shown that indole alkaloids derived from this plant, such as tabernaemontanine and coronaridine achieved 69.34% and 73.40% inhibition of sarcoma 180 at concentrations of 5 µg kg^{-1} day^{-1} and 10 µg kg^{-1} day^{-1}, respectively (Singh *et al.* 2011). Ethanolic extract of this plant demonstrated anticancer potential by damaging the cellular membrane of cancer cells by blocking cellular signaling (Parthiban *et al.* 2015), while ethyl acetate extract effectively inhibited the topoisomerase II in colon cell line (502713) (Thind *et al.* 2008). Additionally, various compounds obtained from this plant, namely coronaridine, voacangine, voacristine, catharanthine, and ibogamine, exhibited a strong binding affinity for human estrogen receptor (Bhagat and Kumar 2021), and crude extracts displayed 50% inhibition of the MCF-7 and MOLT4 cell lines compared to standard drug adriamycin (Doshi *et al.* 2017). Flavonoid fraction from the leaves significantly increased antioxidant level in the liver tissue of EAC and DLAs mouse models reduced liver serum markers, showcasing their significant antioxidant and antitumor effects (Santhi 2020).

Teucrium polium L.:

Teucrium polium, or felty germander, is a shrub with culinary and medicinal uses, containing several phytochemicals such as flavonoids, terpenoids, and iridoids (Bahramikia and Yazdanparast 2012). It has various pharmacological effects ranging from anti-diabetic, antioxidant, and hepatoprotective to anti-microbial, anticancer, and many more (Rahmouni *et al.* 2021). A study by Rajabalian (2008) showed that its extract enhances cytotoxicity and apoptotic effects when combined with vinblastine, vincristine, and doxorubicin, synergistically inhibiting cell growth and inducing apoptosis in human fibroblasts (Rajabalian 2008). Other studies reported that *T. polium* extract inhibits cell proliferation in lung cell lines A-549 and H322 by programmed cell death (Alachkar and Moustafa 2011), and showed significant cytotoxicity in breast and colon cancer cell lines MDA-MB-231 and SW-480 (Nikodijević *et al.* 2016). Research on carcinogenic rats revealed that the decoction from *T. polium* protects liver cells andregulates serum markers, including AFP, ALT, ALP, AST, CBG, HCY, GGT, TNF-α, and α2MG, in a 28-week therapy (Movahedi *et al.*). Additionally, it restored the E-cadherin/catenin complex in prostate cancer cells, reducing metastasis (Kandouz *et al.* 2010). Metabolite profiling identified antioxidant components including dihydrosamidin, cepharantine, 11Z octadecadienoic acid, 13R-hydroxy-9E, and valtratum and *in vitro* studies demonstrated its promising antitumor effects against malignant cell lines MatLyLu and Walker 256/B. Moreover, a docking study demonstrated that these compounds have a strong binding affinity towards the androgen, peroxiredoxin 5, and progesterone receptors (Noumi *et al.* 2020). Methanolic extracts also showed moderate cytotoxic activity against HeLa, MCF-7, and B16F10 cells (Alzeer *et al.* 2015).

Thymus vulgaris carvacrol L.:

This plant is classified in the family *Lamiaceae*. In a recent study, carvacrol derived from this plant significantly enhances cytotoxicity in PC-3 cells via the upregulation of ROS. Furthermore, it suppresses the Notch signaling that leads to G0/G1 cell arrest and reduces the cyclin D1 and Cyclin-Dependent Kinase 4 expression (Khan *et al.* 2019). Another study demonstrated that carvacrol induced apoptosis in osteosarcoma cell lines 1433B and U20S via the Wnt/β-catenin pathway (Zhang *et al.* 2022).

Tillandsia recurvata L.:

Tillandsia recurvata L. (*Bromeliaceae*), is commonly recognized as Old Man's beard or Jamaican Ball Moss. It has been found that *T. recurvata* holds numerous pharmacological properties, including anticancer activity (Lowe *et al.* 2013). Lowe *et al.* 2012 detected the presence of a novel complex glycosidic compound from the supercritical fluid extract, which has been subjected to chromatographic separation followed by spectral analysis, which revealed the potential role of this compound in anticancer activity (Lowe *et al.* 2012). Lowe *et al.* 2017 also highlighted that a flavanone was isolated and showed broad-range anticancer activity

towards MDA-MB-231, brain cancer (U87 MG), neuroblastoma (IMR-32), leukemia (MV4-11), and melanoma (A375), with IC 50 concentrations of 0.030, 0.054, 0.05, 0.024, 0.003 µM, respectively. These findings reveal that *T. recurvata* contains a potent cytotoxic compound that induces apoptosis through the activation of caspase 3/7 (Lowe *et al.* 2017).

***Tribulus terrestris*:**

This plant is a member of the *Zygophyllaceae* family and is commonly found in Africa, America, Asia, and Australia. Its roots and fruits are widely utilized to treat diabetes, liver problems, cancer, and microbial infections (Zhao *et al.* 2023). Studies show that *T. terrestris* extract has promising anticancer effects on MCF-7 and A549 cancer cell lines by inducing apoptosis, enhancing caspase-3 activity, and causing DNA damage, while suppressing Bcl-2 (Allshabi *et al.* 2022). Similarly, another study demonstrated that this plant extract causes apoptosis in the MCF-7 cells through increased levels of *p53* and *Bax* gene expression, DNA damage, and reduced *Bcl-2* gene expression (Patel *et al.* 2019). Naurigenin, a key molecule obtained from *T. terrestris* seeds, also exhibits cytotoxic effects on MCF-7 cells and has significant docking interactions with various receptors like estrogen, progesterone, epidermal growth factor, and epidermal growth factor receptor-2, and apoptotic proteins such as Bax, Bcl-2, caspase-3, -8, and p53 (Patel *et al.* 2021).

***Vitis vinifera* L.:**

Vitis vinifera is the most widely grown and studied grape species globally. Apart from being an economically important source of fruit and wine, it also offers various health benefits (Xu *et al.* 2015). The extracts derived from various parts of *V. vinifera* are known to prevent many pathophysiological situations such as neurodegenerative or cardiovascular diseases, cancers, diabetes, and others (Salehi *et al.* 2019). *V. vinifera* is a rich source of proanthocyanidins, anthocyanins, flavonols, resveratrol, and phenolic acids, of which seed-stored proanthocyanidins show encouraging anticancer and chemopreventive ability in different animal models and cell cultures (Salehi, 2003). Oleanolic acid is present in the ethyl acetate fraction of dried fruit of *V. vinifera*, and it proved to be an efficient therapeutic compound for human colon cancer, showing strong antiproliferative efficacy (Sasikumar *et al.* 2020). In an additional study, antiproliferative activity has been observed on melanoma cell lines (A375 and SK-MEL) (Selma *et al.* 2021). An investigation found that seed extracts from grapes induced apoptosis in the KB cells without a negative impact on the normal cell line HUVEC .

***Withania coagulans* (Stocks) Dunal:**

Withania coagulans is a medicinal plant native to India mentioned in Ayurveda (Mirjalili *et al.* 2009). Historically, this plant has been used for its ethnomedicinal properties, such as immunomodulation, significant antidiabetic properties, hypolipidemic activities, and wound healing effects (Ojha *et al.* 2014). Similarly, the anticancer potential of this plant is also well-documented. The leaf and fruit extract has shown significant cytotoxicity towards human (HeLa, RD, MCF-7) as well as rat (INS-1 and RG2) cancer cell lines (Maqsood *et al.* 2018). Withaferin A, derived from this plant, showed apoptosis in the MDA-MB-231 cells. It showed a decline in cell viability with no noticeable activity observed against normal cell lines (Ahmad *et al.* 2017). Withaferin A showed a cytotoxic effect in KB cells via disruption of cell division at metaphase (Gupta *et al.* 2013). The anticarcinogenic effects of aqueous and methanolic fruit extracts of *W. coagulans* were evaluated using DMBA-induced skin papillomagenesis in Swiss albino mice. The findings indicate that the *W. coagulans* fruit extract effectively prevented tumor formation and reduced tumor incidence, burden, and yield (Mathur and Agrawal 2013). In another study, the plant extract arrested cells in the G2/M phase in MCF7 cells, confirming its efficacy in breast cancer treatment (Gopinath *et al.*2017).

***Withania somnifera* L. Dunal:**

Withania somnifera, commonly known as ashwagandha, is a therapeutic herb widely distributed in India and Africa, known for its various therapeutic benefits, including neuroprotectant, hypoglycemic, hepatoprotection, anti-arthritic, and anticancerous properties (Ghosh 2022). The withanolides in this plant, particularly withaferin A exhibitstrong binding affinity against several proteins of cancer drug targets (Tewari *et al.* 2022), and withanolides can trigger tumor suppressor proteins, limiting cancer cell metastasis (Saggam *et al.* 2020). Studies have shown that *W. somnifera* and its silver nanoparticles have a significant cytotoxic effect on the MCF-7 cancer cells (Prasad *et al.* 2021). A study found that foliar extract exhibited anticancer activity against the various cancer cell lines, including SF-268, MCF-7, NCI-H460, and HCT-116 (Yadav *et al.* 2010). It also demonstrate antitumor and radiosensitizing effects with minimal toxicity in V79 Chinese hamster cells (Devi,1996). The dietary administration of *W. somnifera* roots significantly reduces the formation of forestomach papilloma induced by benzo(a)pyrene and inhibits tumor development. Similar effects have been observed in the case of 7,12-dimethylbenzanthracene-induced skin papilloma,

with a reduction in tumour formation and multiplicity of up to 45 and 71%, respectively (Padmavathi *et al.* 2005). Withaferin A shows strong growth inhibition and cytotoxicity against NCI-H460 cells (Choudhary *et al.* 2010). Additionally, 16β-acetoxy-6α,7α-epoxy-5α-hydroxy-1-oxowitha-2,17(20), 24-trienolide isolated from leaves exhibited significant cytotoxic effects on WRL-68, MCF-7, Caco-2, and PC-3 cell lines (Siddique *et al.* 2014). *W. somnifera* demonstrates promising anticancer and immunomodulating properties, indicating its potential as an effective anticancer drug.

***Zea mays* L.:**

Zea mays, traditionally used in China, France, Turkey, etc., is rich in maysin, a maize-specific flavonoid (Wrigley *et al.* 2015). The anticancer activity of maysin derived from corn silk of *Z. mays* L. was investigated using PC-3 cells. Maysin treatment significantly reduced cell survival by apoptosis induction, DNA damage, and reduced the transcription of pro-caspase-3 and Bcl-2 in PC-3 cells. It also attenuates the Akt and ERK phosphorylation (Lee *et al.* 2014). A study demonstrated the shielding efficacy of maize silk extract in mice against oxidative stress induced by radiation (Bai *et al.* 2010). Yet another report proves corn silk extract inhibits cell proliferation in the LoVo cell line (Guo *et al.* 2017) and also shows the apoptotic effect in C6 rat glioma cells via the generation of ROS (Hwang *et al.* 2018). The mechanistic action of corn silk extract has been proved by assessment of the apoptotic marker gene expression and ROS generation in MCF-7 cells (Al-Oqail *et al.* 2019). Leaf extract of this plant was also assessed to check its influence on the apoptosis process triggered by hydrogen peroxide in Hep-2 cells. It has been observed that aqueous and methanol extracts demonstrated cytotoxicity similar to the H_2O_2-treated groups on oxidatively stressed cancer cells (Balasubramanian and Padma 2013).

***Zingiber officinale* Roscoe:**

This plant is classified under the family *Zingiberaceae* and is popularly known as ginger. Methanolic extract of zingiber rhizome significantly inhibits cell proliferation in MDA-MB-231 cells depending on the dosage (Park *et al.* 2014). Extract of ginger induced apoptosis via increasing Bax and downregulated the transcripts of *Bcl-X, Bcl-2, cyclin D1, NF-κB, and CDK-4* in MDA-MB-231 and MCF-7 cell lines. Rhizome extract of *Z. officinale* inhibits the proliferation in HeLa and MDA-MB-231 cell lines (Ansari *et al.* 2016).

References

Abomughaid, M.M., Teibo, J.O., Akinfe, O.A., Adewolu, A.M., Teibo, T.K.A., Afifi, M., Al-Farga, A.M.H., Al-kuraishy, H.M., Al-Gareeb, A.I., Alexiou, A. and Papadakis, M., 2024. A phytochemical and pharmacological review of Ricinus communis L. Discover Applied Sciences, 6(6), p.315.

Aghbali, A., Hosseini, S.V., Delazar, A., Gharavi, N.K., Shahneh, F.Z., Orangi, M., Bandehagh, A. and Baradaran, B., 2013. Induction of apoptosis by grape seed extract (Vitis vinifera) in oral squamous cell carcinoma. Bosn. J. Basic Med. Sci. 13(3), 186.

Ahmad, R., Fatima, A., Srivastava, A.N. and Khan, M.A., 2017. Evaluation of apoptotic activity of Withania coagulans methanolic extract against human breast cancer and Vero cell lines. J. Ayurveda Integr. Med. 8(3), 177-183.

Ahmed, Y., Akhtar, P., Rahman, S. and Yaakob, Z., 2016. Chemical constituents of Saurauia roxburghii. Chem. Nat. Compd. 52, 953-955.

Alachkar, A. and Al Moustafa, A.E., 2011. Teucrium polium plant extract provokes significant cell death in human lung cancer cells. Health, 3(06), p.366.

Al-Oqail, M.M., Al-Sheddi, E.S., Farshori, N.N., Al-Massarani, S.M., Al-Turki, E.A., Ahmad, J., Al-Khedhairy, A.A. and Siddiqui, M.A., 2019. Corn silk (Zea mays L.) induced apoptosis in human breast cancer (MCF-7) cells via the ROS-mediated mitochondrial pathway. Oxid. Med. Cell. Longev. 2019.

Alshabi, A.M., Alkahtani, S.A., Shaikh, I.A., Orabi, M.A., Abdel-Wahab, B.A., Walbi, I.A., Habeeb, M.S., Khateeb, M.M., Shettar, A.K. and Hoskeri, J.H., 2022. Tribulus terrestris Cytotoxicity against Breast Cancer MCF-7 and Lung Cancer A549 Cell Lines Is Mediated via Activation of Apoptosis, Caspase-3, DNA Degradation, and Suppressing Bcl-2 Activity. Separations, 9(11), p.383.

Alzeer, J., Vummidi, B.R., Arafeh, R., Rimawi, W., Saleem, H. and Luedtke, N.W., 2015. The influence of extraction solvents on the anticancer activities of Palestinian medicinal plants.

Ansari, J.A., Ahmad, M.K., Khan, A.R., Fatima, N., Khan, H.J., Rastogi, N., Mishra, D.P. and Mahdi, A.A., 2016. Anticancer and antioxidant activity of Zingiber officinale Roscoe rhizome. Indian J. Exp. Biol. 54, 767- 773.

Bahramikia, S. and Yazdanparast, R., 2012. Phytochemistry and medicinal properties of Teucrium polium L. (Lamiaceae). Phytother. Res. 26(11), 1581-1593.

Bai, H., Hai, C., Xi, M., Liang, X. and Liu, R., 2010. Protective effect of maize silks (Maydis stigma) ethanol extract on radiation-induced oxidative stress in mice. Plant Foods Hum. Nutr. 65(3), 271-276.

Balasubramanian, K. and Padma, P.R., 2013. Anticancer activity of Zea mays leaf extracts on oxidative stress-induced Hep2 Cells. J. Acupunct. Meridian Stud. 6(3), 149-158.

Bhagat, S. and Kumar, D., 2021. A recepto-informatics study of the phytocompounds of Tabernaemontana divaricata (L.) having potential anticancer efficacy for breast cancer. J. Med. Plant Res. 9(1), 14-20.

Choudhary, M.I., Hussain, S., Yousuf, S. and Dar, A., 2010. Chlorinated and diepoxy withanolides from Withania somnifera and their cytotoxic effects against human lung cancer cell line. Phytochemistry, 71(17-18), 2205-2209.

Devi, P.U., 1996. Withania somnifera Dunal (Ashwagandha): potential plant source of a promising drug for cancer chemotherapy and radiosensitization. Indian J. Exp. Biol. 34(10), 927-932.

Doshi, G.M., Kanad, P.P., Azad, N., Desai, A., Somani, R.R. and Chaskar, P.K., 2017. In vitro Cytotoxicity Studies on Tabernaemontana divaricata leaves extracts by sulforhodamine B assay method. Int. J. Pharm. Sci. Rev. Res, 45, 179-182.

Fabelico, F., 2020. Phytochemical, antioxidant, cytotoxic, and antimicrobial properties of the endemic dagwey (Saurauia subglabra Merr.) of the Philippines. J. Crit. Rev.7(11).

Ghosh, T., 2022. Ashwagandha: a medicinal plant in the context of Indian medicine treatment. Int. J. Phytol. Res. 2(2),18-21.

Gopinath, S.M., Ismail Shareef, M., Gupta, S. and Gupta, A., 2017. In vitro anticancer evaluation of Withania coagulans on breast cancer cell lines. World J. Pharm. Pharm. Sci. 6(9), 933- 940.

Guo, H., Guan, H., Yang, W., Liu, H., Hou, H., Chen, X., Liu, Z., Zang, C., Liu, Y. and Liu, J., 2017. Pro-apoptotic and anti-proliferative effects of corn silk extract on human colon cancer cell lines. Oncol. Lett. 13(2), 973-978.

Gupta, V. and Keshari, B.B., 2013. Withania coagulans Dunal (paneer doda): A review. Int. J. Ayurvedic Herb. Med. 3(5), 1330-1336.

Hwang, E., Sim, S., Park, S.H., Song, K.D., Lee, H.K., Heo, T.H., Jun, H.S. and Kim, S.J., 2018. Anti-proliferative effect of Zea mays L. cob extract on rat C6 glioma cells through regulation of glycolysis, mitochondrial ROS, and apoptosis. Biomed. Pharmacother. 98, 726-732.

Jeenathunisa, N. and Rajan, S., 2021. In vitro Cytotoxic studies of Saraca asoca bark extracts on HT-29 cancer cell Line. Res. J. Pharm. Technol. 14(1), 42-46.

Jinu, M.V. and Jayabaskaran, C., 2015. Diversity and anticancer activity of endophytic fungi associated with the medicinal plant Saraca asoca. Curr. Res. Environ. Appl. Mycol. 5, 169-179.

Kandouz, M., Alachkar, A., Zhang, L., Dekhil, H., Chehna, F., Yasmeen, A. and Al Moustafa, A.E., 2010. Teucrium polium plant extract inhibits cell invasion and motility of human prostate cancer cells via the restoration of the E-cadherin/catenin complex. J. Ethnopharmacol. 129(3), 410-415.

Khan, F., Singh, V.K., Saeed, M., Kausar, M.A. and Ansari, I.A., 2019. Carvacrol induced program cell death and cell cycle arrest in androgen-independent human prostate cancer cells via inhibition of notch signaling. Anti-Cancer Agents Med. Chem. 19(13), 1588-1608.

Kumar, A., Sreedharan, S., Kashyap, A.K., Singh, P. and Ramchiary, N., 2022. A review on bioactive phytochemicals and ethnopharmacological potential of purslane (Portulaca oleracea L.). Heliyon, 8(1).

Lee, J., Lee, S., Kim, S.L., Choi, J.W., Seo, J.Y., Choi, D.J. and Park, Y.I., 2014. Corn silk maysin induces apoptotic cell death in PC-3 prostate cancer cells via mitochondria-dependent pathway. Life Sci. 119(1-2), 47-55.

Lee, M.T., Chang, C.I., Lin, J.J. and Wu, Y.J., 2022. Piper betle stem extract induces apoptosis through mitochondria inactivation, p38MAPK activation and ErB2/ErbB3 suppression on oral squamous cell carcinoma cells. Phytomedicine Plus, 2(3), p.100295.

Looi, M.L., Wong, A.K.H., Gnapragasan, S.A., Japri, A.Z., Rajedadram, A. and Pin, K.Y., 2020. Anti-migratory effects of Piper betle leaf aqueous extract on cancer cells and its microtubule targeting properties. Journal of Zhejiang University. Science. B, 21(9), p.745.

Lowe, H., Toyang, N. and Bryant, J., 2013. In vitro and In vivo anti-cancer effects of Tillandsia recurvata (Ball Moss) from Jamaica. West Indian Med. J. 62(3), 177-80.

Lowe, H.I., Toyang, N.J., Watson, C.T., Ayeah, K.N. and Bryant, J., 2017. HLBT-100: a highly potent anti-cancer flavanone from Tillandsia recurvata (L.) L. Cancer Cell Int. 17(1), 1-12.

Lowe, H.I., Watson, C.T., Badal, S., Toyang, N.J. and Bryant, J., 2012. Kinase inhibition by the Jamaican ball moss, Tillandsia recurvata L. Anticancer Res. 32(10), 4419-4422.

Majumder, M., Debnath, S., Gajbhiye, R.L., Saikia, R., Gogoi, B., Samanta, S.K., Das, D.K., Biswas, K., Jaisankar, P. and Mukhopadhyay, R., 2019. Ricinus communis L. fruit extract inhibits migration/invasion, induces apoptosis in breast cancer cells and arrests tumor progression in vivo. Scientific Reports, 9(1), p.14493.

Maqsood, M., Qureshi, R., Ikram, M., Ahmad, M.S., Jabeen, B., Asi, M.R., Khan, J.A., Ali, S. and Lilge, L., 2018. In vitro anticancer activities of Withania coagulans against HeLa, MCF-7, RD, RG2, and INS-1 cancer cells and phytochemical analysis. Integr. Med. Res. 7(2), 184-191.

Mathur, D. and Agrawal, R.C., 2013. Anticarcinogenic potential of Withania coagulans fruit against skin papilomagenesis in Swiss albino mice. Rec. Res. Sci. Technol, 5, 1-4.

Mirjalili, M.H., Moyano, E., Bonfill, M., Cusido, R.M. and Palazón, J., 2009. Steroidal lactones from Withania somnifera, an ancient plant for novel medicine. Molecules, 14(7), 2373-2393.

Movahedi, A., Basir, R., Rahmat, A., Charaffedine, M. and Othman, F., 2014. Remarkable anticancer activity of Teucrium polium on hepatocellular carcinogenic rats. Evidence-Based Complementary and Alternative Medicine, 2014(1), p.726724.

Nahrin, A., Junaid, M., Afrose, S.S., Alam, M.S., Hosen, S.M., Akter, R. and Sharmin, T., 2020. A review of Saurauia roxburghii Wall. (Actinidiacaea) as a traditional medicinal plant, its phytochemical study and therapeutic potential. Mini Rev. Med. Chem. 20(19), 2036-2051.

Nikodijević, D., Milutinović, M., Cvetković, D., Stanković, M., Živanović, M.N. and Marković, S., 2016. Effects of Teucrium polium L. and Teucrium montanum L.: Extracts on mechanisms of apoptosis in breast and colon cancer cells. Kragujev. J. Sci. 38, 147-159.

Noumi, E., Snoussi, M., Anouar, E.H., Alreshidi, M., Veettil, V.N., Elkahoui, S., Adnan, M., Patel, M., Kadri, A., Aouadi, K. and De Feo, V., 2020. HR-LCMS-based metabolite profiling, antioxidant, and anticancer properties of Teucrium polium L. methanolic extract: Computational and in vitro study. Antioxidants. 9(11), 1089.

Ojha, S., Alkaabi, J., Amir, N., Sheikh, A., Agil, A., Fahim, M.A. and Adem, A., 2014. Withania coagulans fruit extract reduces oxidative stress and inflammation in kidneys of streptozotocin-induced diabetic rats. Oxid. Med. Cell. Longev. 2014.

Padmavathi, B., Rath, P.C., Rao, A.R. and Singh, R.P., 2005. Roots of Withania somnifera inhibit forestomach and skin carcinogenesis in mice. Evid. Based Complementary Altern. Med. 2(1), 99-105.

Pareeth, C.M., Hussan, K.S., Anu, D., Meera, N., Mathew, D., Valsalan, R., Thayyil, M.S., Thara, K.M., Raghavamenon, A.C. and Babu, T.D., 2024. Antiproliferative effect of Saraca asoca methanol bark extract on triple negative breast cancer (TNBC). Future Journal of Pharmaceutical Sciences, 10(1), p.52.

Park, G.H., Park, J.H., Song, H.M., Eo, H.J., Kim, M.K., Lee, J.W., Lee, M.H., Cho, K.H., Lee, J.R., Cho, H.J. and Jeong, J.B., 2014. Anti-cancer activity of Ginger (Zingiber officinale) leaf through the expression of activating transcription factor 3 in human colorectal cancer cells. BMC Complement Altern. Med.14, 1-8.

Parthiban, S.U.J.I.T.H.A., Raman, R., Muthuraman, M.S. and Pemaiah, B., 2015. Antitumor potential of Ervatamia divaricata. L. Burkill against Ehrlich ascites carcinoma. Int. J. Pharm. Pharm. Sci. 7(7), 171-175.

Patel, A., Bhatt, M., Soni, A. and Sharma, P., 2021. Identification of steroidal saponins from Tribulus terrestris and their in silico docking studies. Journal of Cellular Biochemistry, 122(11), pp.1665-1685.

Patel, A., Soni, A., Siddiqi, N.J. and Sharma, P., 2019. An insight into the anticancer mechanism of Tribulus terrestris extracts on human breast cancer cells. 3 Biotech, 9(2), p.58.

Prasad, K.S., Prasad, S.K., Veerapur, R., Lamraoui, G., Prasad, A., Prasad, M.N., Singh, S.K., Marraiki, N., Syed, A. and Shivamallu, C., 2021. Antitumor potential of green synthesized ZnONPs using root extract of Withania somnifera against human breast cancer cell line. Separations, 8(1), 8.

Rahimi, V.B., Mousavi, S.H., Haghighi, S., Soheili-Far, S. and Askari, V.R., 2019. Cytotoxicity and apoptogenic properties of the standardized extract of Portulaca oleracea on glioblastoma multiforme cancer cell line (U-87): a mechanistic study. EXCLI journal, 18, p.165.

Rahmouni, F., Saoudi, M. and Rebai, T., 2021. Therapeutics studies and biological properties of Teucrium polium (Lamiaceae). Biofactors, 47(6), 952-963.

Rajabalian, S., 2008. Methanolic extract of Teucrium polium L potentiates the cytotoxic and apoptotic effects of anticancer drugs of vincristine, vinblastine and doxorubicin against a panel of cancerous cell lines. Experimental Oncology. 30(2), 133-8.

Roy, A., Lakshmi, T., Raghunandhakumar, S., Elumalai, P. and Sarojini, K., 2021. Evaluation of Proapoptotic Potential of Saraca asoca Flower Extract on Skin Cancer Cell Line. J. pharm. res. Int. 436-446.

Saggam, A., Tillu, G., Dixit, S., Chavan-Gautam, P., Borse, S., Joshi, K. and Patwardhan, B., 2020. Withania somnifera (L.) Dunal: A potential therapeutic adjuvant in cancer. J. Ethnopharmacol. 255, 112759.

Salehi, B., Vlaisavljevic, S., Adetunji, C.O., Adetunji, J.B., Kregiel, D., Antolak, H., Pawlikowska, E., Uprety, Y., Mileski, K.S., Devkota, H.P. and Sharifi-Rad, J., 2019. Plants of the genus Vitis: Phenolic compounds, anticancer properties and clinical relevance. Trends Food Sci. Technol. 91, 362-379.

Santhi, R., 2020. Preliminary evaluation of in vitro and in vivo antioxidative and antitumor activities of flavonoid extract of Tabernaemontana divaricata leaves in Ehrlich's lymphoma and Dalton's lymphoma ascites model. 8 J. Cancer Res. Ther. 16(1), 78-87.

Sasikumar, K., Dubey, V. and Ghosh, A.R., 2020. Oleanolic acid from black raisins, Vitis vinifera with antioxidant and antiproliferative potentials on HCT 116 colon cancer cell line. Braz. J. Pharm. Sci. 56.

Selma, F., Chahinez, T., Salim, G. and Sakina, Z., 2021. Antioxidant and Anti-cancer effects of crude extracts from (Vitis vinifera L.) leaves on melanoma cells (SK-Mel and A375). Emir. J. Food Agric. 691-698.

Siddique, A.A., Joshi, P., Misra, L., Sangwan, N.S. and Darokar, M.P., 2014. 5, 6-De-epoxy-5-en-7-one-17-hydroxy withaferin A, a new cytotoxic steroid from Withania somnifera L. Dunal leaves. Nat. Prod. Res. 28(6), 392-398.

Singh, B., A Sharma, R. and K Vyas, G., 2011. Antimicrobial, antineoplastic and cytotoxic activities of indole alkaloids from Tabernaemontana divaricata (L.) R. BrCurr. Pharm. Anal. 7(2), 125-132.

Surh, Y.J., 2003. Cancer chemoprevention with dietary phytochemicals. Nat. Rev. Cancer. 3(10), 768-780.
Tewari, D., Chander, V., Dhyani, A., Sahu, S., Gupta, P., Patni, P., Kalick, L.S. and Bishayee, A., 2022. Withania somnifera (L.) Dunal: Phytochemistry, structure-activity relationship, and anticancer potential. Phytomedicine. 153949.
Thind, T.S., Agrawal, S.K., Saxena, A.K. and Arora, S., 2008. Studies on cytotoxic, hydroxyl radical scavenging and topoisomerase inhibitory activities of extracts of Tabernaemontana divaricata (L.) R. Br. ex Roem. and Schult. Food Chem. Toxicol. 46(8), 2922-2927.
Vinusri, S., Gnanam, R., Caroline, R., Santhanakrishnan, V.P. and Kandavelmani, A., 2022. Anticancer potential of hydroxychavicol derived from piper betle L: An in silico and cytotoxicity study. Nutrition and Cancer, 74(10), pp.3701-3713.
Wrigley, C.W., Corke, H., Seetharaman, K. and Faubion, J. eds., 2015. Encyclopedia of food grains. Academic Press.
Xu, C., Yagiz, Y., Marshall, S., Li, Z., Simonne, A., Lu, J. and Marshall, M.R., 2015. Application of muscadine grape (Vitis rotundifolia Michx.) pomace extract to reduce carcinogenic acrylamide. Food Chem. 182, 200-208.
Yadav, B., Bajaj, A., Saxena, M. and Saxena, A.K., 2010. In vitro anticancer activity of the root, stem and leaves of Withania somnifera against various human cancer cell lines. Indian J. Pharm. Sci. 72(5), 659.
Yadav, N.K., Saini, K.S., Hossain, Z., Omer, A., Sharma, C., Gayen, J.R., Singh, P., Arya, K.R. and Singh, R.K., 2015. Saraca indica bark extract shows in vitro antioxidant, antibreast cancer activity and does not exhibit toxicological effects. Oxid. Med. Cell. Longev. 2015.
Zhang, S., He, L., Shang, J., Chen, L., Xu, Y., Chen, X., Li, X., Jiao, Q., Jin, S., Hu, X. and Liang, W., 2022. Carvacrol Suppresses Human Osteosarcoma Cells via the Wnt/β-Catenin Signaling Pathway. Anticancer Agents Med. Chem. 22(9), 1714-1722.
Zhao, C., Ying, Z., Tao, X., Jiang, M., Ying, X. and Yang, G., 2018. A new lactam alkaloid from Portulaca oleracea L. and its cytotoxity. Natural product research, 32(13), pp.1548-1553.
Zhao, J., Tian, X.C., Zhang, J.Q., Li, T.T., Qiao, S. and Jiang, S.L., 2023. Tribulus terrestris L. induces cell apoptosis of breast cancer by regulating sphingolipid metabolism signaling pathways. Phytomedicine, 120, p.155014.
